# The Effect of Mechanical Circulatory Support on Blood Flow in the Ascending Aorta: A Combined Experimental and Computational Study

**DOI:** 10.3390/bioengineering11030238

**Published:** 2024-02-28

**Authors:** Sapir Hazan Shenberger, Idit Avrahami

**Affiliations:** Department of Mechanical Engineering and Mechatronics, Ariel University, Ariel 40700, Israel

**Keywords:** percutaneous MSC, aortic flow, TKE, PIV, CFD

## Abstract

Percutaneous mechanical circulatory support (MCS) devices are designed for short-term treatment in cases of acute decompensated heart failure as a bridge to transplant or recovery. Some of the known complications of MCS treatments are related to their hemodynamics in the aorta. The current study investigates the effect of MCS on the aortic flow. The study uses combined experimental and numerical methods to delineate complex flow structures. Particle image velocimetry (PIV) is used to capture the vortical and turbulent flow characteristics in a glass model of the human aorta. Computational fluid dynamics (CFD) analyses are used to complete the 3D flow in the aorta. Three specific MCS configurations are examined: a suction pump with a counterclockwise (CCW) rotating impeller, a suction pump with a clockwise (CW) rotating impeller, and a discharge pump with a straight jet. These models were examined under varying flow rates (1–2.5 L/min). The results show that the pump configuration strongly influences the flow in the thoracic aorta. The rotating impeller of the suction pump induces a dominant swirling flow in the aorta. The swirling flow distributes the incoming jet and reduces the turbulent intensity near the aortic valve and in the aorta. In addition, at high flow rates, the local vortices formed near the pump are washed downstream toward the aortic arch. Specifically, an MCS device with a CCW rotating impeller induces a non-physiological CCW helical flow in the descending aorta (which is opposite to the natural helical flow), while CW swirl combines better with the natural helical flow.

## 1. Introduction

In the past decade, the management of severe acute heart failure (AHF) has been significantly transformed by the introduction of percutaneously implanted mechanical circulatory support (MCS) devices [1]. These MCS devices, inserted via a catheter, play a crucial role in temporarily maintaining blood flow during high-risk procedures, such as percutaneous coronary intervention (PCI), transcatheter aortic valve replacement (TAVR), and balloon aortic valvuloplasty (BAV), especially in cases involving cardiogenic shock and severe left ventricle (LV) dysfunction [2,3]. They act as a bridge to recovery (or bridge to decision), facilitating blood flow to vital organs while alleviating strain on the recovering myocardium and allowing the LV to rest. MCS devices augment systemic cardiac output by creating continuous blood flow from the LV cavity into the ascending aorta [4].

Percutaneous MCS devices are positioned between the LV and the aorta, passing through the aortic valve. These devices can be categorized based on the impeller’s location within the pump relative to the LV and the aorta. They are either distal, situated in the aorta and drawing blood from the LV through a tube [5], or proximal, located inside the LV and directing blood from the LV to the aorta [6].

However, these devices are not without complications. Designing these devices presents significant engineering challenges due to the constraints on catheter sizes. One of the main challenges associated with miniature MCS devices is the need for increased angular speed to meet the high demands for sufficient flow and pressure [7,8,9]. Despite their hemodynamic effectiveness [1], the clinical use of MCS is accompanied by several major complications and long-term side effects. These include device thrombosis, both in the form of pump thrombosis and thromboembolic events, as well as bleeding, hemolysis, device malfunction, strokes, anemia, and significant impacts on morbidity and overall mortality [10,11,12]. A significant proportion of patients may develop a progression of aortic valve regurgitation or aortic valve insufficiency [13,14]. Some of these complications are attributed to the non-physiological flow induced by the device in the aorta and its impact on the aortic flow [15].

The natural flow in the aorta is dominated by helical flow, attributed to the curvature of the aorta’s geometry. This helical flow is accompanied by secondary flow vortices that are attributed to the non-planar tapering aorta geometry and the boundary conditions of ventricular twisting and the aortic valve [16,17,18]. The helical flow in the aorta is defined as a corkscrew-like flow of blood [19]. The helical flow is assumed to facilitate ventricular ejection, promote oxygen transfer, and reduce the concentration of low-density lipoproteins on the luminal surface [20]. This normal degree of swirl is hypothesized to stabilize aortic flow patterns and reduce turbulence [21].

However, high values of turbulent kinetic energy (TKE) in the aortic flow are correlated with a variety of vascular dysfunction mechanisms, such as reduction in nitric oxide (NO) production, endothelial cell activation, and platelet adhesion, leading to vascular pathologies [22,23,24,25]. Turbulence increases the fluid dynamic shear stress on blood constituents and promotes platelet aggregation, leading to thrombus development in disturbed flow regions and damage to red blood cells [24,26,27]. Turbulent and vortical flow in the aorta is also associated with aneurysm formation [28].

Gulan et al. [29] investigated a 3D pulsatile flow in an in vitro realistic compliant model of a human ascending aorta using the 3D-PIV technique. They showed that the velocity profile at the inlet of the ascending aorta is relatively flat with a skewed profile toward the inner aortic wall.

Although the helical flow in a healthy aorta was proven to have advantages, when it comes to swirling flow emerging from implanted left ventricle assist devices (LVADs), it may significantly influence the flow in the aortic hemodynamics, and therefore, it is necessary to consider the effects of the swirling component of LVAD outflow in LVAD design [30,31,32].

It was suggested that similar to the natural swirling blood flow in the human aorta, swirling flow caused by an axial LVAD could provide favorable conditions to maintain aortic function by providing the inner surface of the ascending aortic wall with specific hemodynamic features that promote its smoothness, enable proper washout, and prevent atherosclerotic plaques from forming [33]. The specific flow patterns that occur in the vicinity of the aortic sinus and the ascending aorta due to the emerging continuous swirling jet may reduce the concentration of low-density lipoprotein (LDL) in the luminal surface of the aortic arch and play roles in suppressing severe atherosclerosis and regulating vascular smooth muscle cell function. It was suggested, in this respect, that a series-type LVAD may promote the benefits of swirling flow. However, when the LVAD’s rotating direction is in the opposite direction of the natural helical flow, it may lead to the remodeling of the aorta, but its precise effect is unclear [33].

Although many studies have used experimental methods (in vivo and in vitro) to study the flow in a healthy aorta [17,34,35] and those with pathologies [36,37] or implanted grafts [38], most of the previous studies on MCS flow have focused mainly on the flow inside the pump and through the pump’s impeller [39,40,41,42]. Very few studies have used numerical simulation to study the flow in an aorta in the presence of an implanted LVAD [30,31,32,33,43]. Wang et al. [32] presented a quantitative CFD simulation of the hemodynamic effects of the Impella^TM^ MCS device in a model of the aorta to demonstrate that the Impella CP support augmented the velocity, WSS, and pressure drop within the aorta. Wang et al. [32] used CFD analysis to describe the additional effect of MCS on the native pulsating aortic flow and the wall shear stress distribution for a patient-specific aorta model.

To the best of our knowledge, no experimental study investigating the impact of various MCS configurations on aortic flow has been conducted thus far. Furthermore, the specific issue concerning aortic hemodynamics associated with turbulent and helical flow in the presence of MCS remains unexplored. In the present study, our focus is directed toward examining the flow downstream of the MCS device in the thoracic aorta. We employed a combination of experimental and numerical methods to elucidate the influence of the emerging swirling jet on aortic hemodynamics, turbulence, and vortices under different flow and design configurations.

## 2. Methods and Materials

We investigated the hemodynamic behavior of flow in a planar model of the aorta in the presence of an MCS device to capture the flow characteristics and establish correlations between the vorticity and turbulence parameters in the flow field with the design and flow conditions of the pump. The analysis was conducted using a combination of two methods: particle image velocimetry (PIV) and computational fluid dynamics (CFD). While the flow in the aorta is inherently three-dimensional (3D), the PIV method provides a 2D projection of the flow onto an examined plane. Thus, the numerical simulation complements the 3D perspective.

We explored three pump configurations and their impact on the flow in the aorta: a discharge pump (jet inlet case) and a suction pump with a rotating impeller in both the clockwise (CW) and counterclockwise (CCW) directions.

Firstly, we validated the numerical model using experiments with four different flow rates ranging from 1 to 2.5 L per minute (L/min) for each pump configuration. During the validation, we assumed that the flow emerging from the MCS device was constant and that the heart was still, neglecting the effects of heartbeats and wall compliance. Additionally, we neglected the flow toward the upper vessels. Next, we utilized the numerical model to explore cases with higher flow rates where the constant flow from the pump was combined with the pulsating flow from the heart.

### 2.1. Experimental Setup

**Figure 1:**
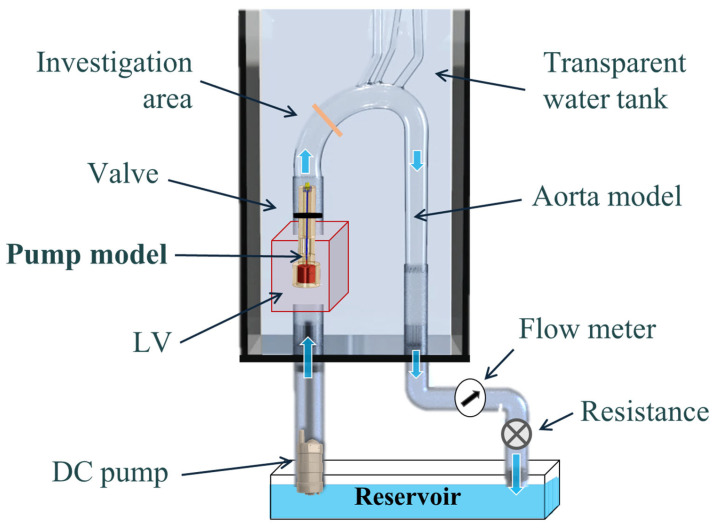
A schematic description of the hydraulic experimental setup, including a glass model, two pumps, a reservoir, a regulating valve, and sealing rubber. Blue arrows indicate flow direction.

**Figure 2:**
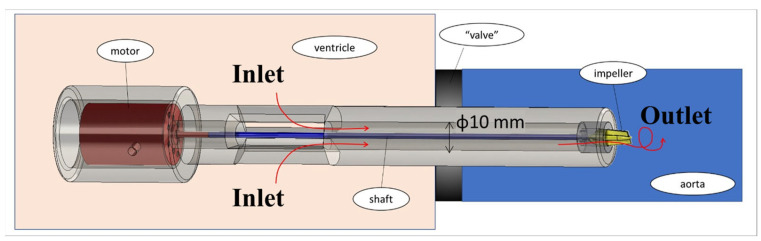
The experimental model of the MCS rotary pump. Red arrows indicate flow direction.

The experimental system is described in Figure 1. The basic components of the hydraulic system included an aortic glass model and a closed flow loop derived from the pump model. The aortic glass model, previously employed in our research [44], possesses typical anatomical geometry and dimension [45,46]. A closed container positioned proximal to the model was used as the LV, while a rubber seal modeled a closed valve. The experimental model of the MCS pump (Figure 2) incorporated a motor, housing, rotating shaft, and an impeller located at the end of the shaft. Rotation of the shaft induced a swirling flow at the proximal inlet of the aorta. The flow rate of each case (as defined in Table 1) was regulated by a combination of a regulating valve and a submersible DC bypass pump, with a measurement conducted using a calibrated rotameter (DN 15, KONGCHENG Fittings, Wenzhou, China). Specifically, the DC pump acted as a “discharge pump” in the case of the “jet inlet” (providing the required forward flow while the impeller pump did not run), and as a secondary auxiliary pump in the CCW case to reduce the required pump head (which otherwise resulted in lower flow rates than in the CW case).

To replicate the properties of blood, we employed a transparent solution consisting of water and glycerol with a volumetric ratio of 2 parts water to 1 part glycerol. The viscosity of the solution was determined before and after each experiment using an Ostwald viscometer and ranged from μ = 3.5 to 4.0 cP. Uncoated neutrally buoyant hollow glass spheres with a nominal diameter of 11 μm were used as seeding particles (Potters Sphericel). To minimize laser light scattering and optical distortion due to reflections from the curved glass model’s wall, the model was immersed in a water-filled tank made of acrylic glass (PMMA).

**Figure 3:**
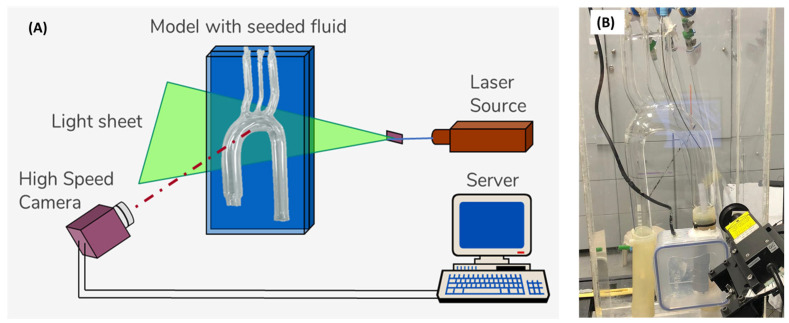
The PIV system: (**A**) schematic description of the PIV setup, and (**B**) photo of the experimental model.

The PIV system (Figure 3) comprised a high-speed CMOS monochromatic camera (2320 × 1726 pixels, Bonito CL 400, Allied Vision, Stadtroda, Germany) with a precise internal shutter clock and timing control. It was positioned at approximately 35 cm in front of the model, at a 90º angle to a continuous-wave laser light sheet (MGL-III, 200 mW, 532 nm, CNI). To capture the images, a Zeiss Distagon T* 35 mm f/2.0 ZF.2 lens was attached to the camera, providing a resolution of 0.06 mm/pixel. The camera was connected to a server (DELL PowerEdge T710) equipped with a PIXCI^®^ frame grabber and Xcap^TM^ software for real-time image acquisition. The frame rate was set at 192 Hz, and the PIV algorithm was configured with a 50% overlap. In addition to the minimal effect of image distortion (due to the curved walls of the model), the post-processing algorithm used a pre-defined calibrated image mask of the model, as described in a previous study [44], which used the same model and was well-calibrated for optical distortions.

### 2.2. Cases Studied

**Figure 4:**
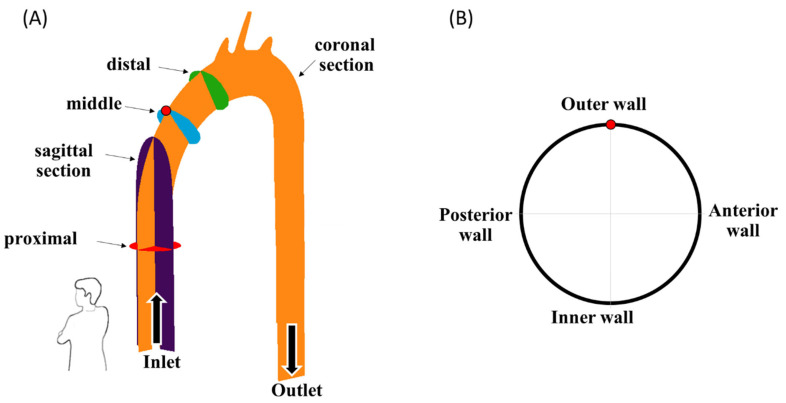
(**A**) The examined cross-sections, and (**B**) an example orientation view of the blue vertical cross-sections. Arrows indicate flow direction.

Three different MCS configurations were examined: *CW inlet* (a “suction pump” case with an impeller that rotates in the CW direction), *CCW inlet* (a “suction pump” case with an impeller that rotates in the CCW direction), and *jet inlet* (a “discharge pump” case in which the inlet flow to the aorta is mostly straight, with neglected radial components). In each case, four different flow rates of 1 L/min, 1.5 L/min, 2 L/min, and 2.5 L/min were examined (as listed in Table 1). For each case, five different cross-sections were recorded (Figure 4A): two cross-sections parallel to the flow direction—a coronal central plane (orange in the figure) and a sagittal central plane (purple), and three cross-sections orthogonal to the flow along the ascending aorta—a proximal (red), middle (blue), and distal section (green). The results of the three vertical cross-sections (proximal, middle, and distal) are presented in orientation in Figure 4B. Altogether, 12 cases were examined, with 5 sections each. For each case and section, 990 frames were recorded at a frame rate of 192 Hz, resulting in 989 PIV velocity fields (using 50% overlap).

### 2.3. Post-Processing

The open-source PIVlab software (version 2.59) [47,48] was used to analyze the recorded images, with the multi-pass FFT cross-correlation algorithm using interrogation windows of 64 × 64 and 32 × 32 pixels, with a 50–120 μm/pixel resolution (depending on the specific cross-section). The data were imported, post-processed, and analyzed using the MATLAB open-source toolbox PIVMAT [49] and using MATLAB (MathWorks, Natick, MA, USA, R2021) to calculate components of vorticity and TKE. The images were calibrated and converted into “true world” coordinates using a known calibration grid of distances.

After calculating the PIV velocity field for each case and section, the vorticity vector ($\vec{\omega }$) perpendicular to the plane and the TKE values were calculated using the following expressions:

$$\omega_{z}=\frac{𝜕u}{𝜕x}-\frac{𝜕v}{𝜕y}$$

$$TKE=\frac{1}{2}\left({\left(\frac{1}{T}\int_{0}^{T}\left|u\left(t\right)-u\right|dt\right)}^{2}+{\left(\frac{1}{T}\int_{0}^{T}\left|v\left(t\right)-v\right|dt\right)}^{2}\right)$$

where *T* is the total duration of the recorded session, *u*(*t*) and *v*(*t*) are the *x* and *y* velocity components of the 2D instantaneous velocity vector, and *u* and *v* are the mean velocity components averaged over all the *n* = 989 velocity maps:

$$u=\frac{1}{T}\int_{0}^{T}u\left(t\right)dt=\frac{1}{n}\sum_{i=1}^{n}{u(t}_{i}),v=\frac{1}{T}\int_{0}^{T}v\left(t\right)dt=\frac{1}{n}\sum_{i=1}^{n}{v(t}_{i})$$

### 2.4. Numerical Model

**Figure 5:**
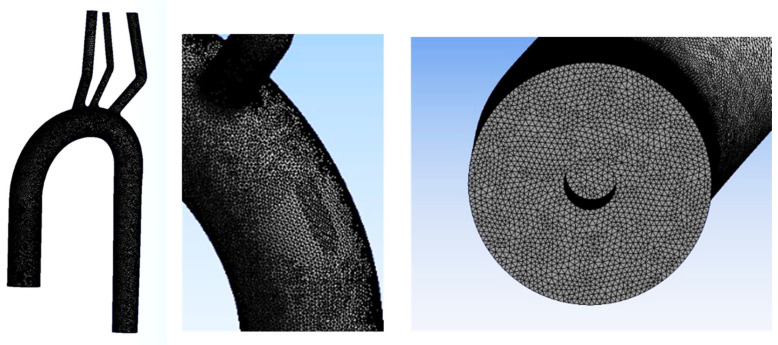
The numerical mesh.

The simulations used CFD methods to delineate the steady-state flow in the model. The commercial software Fluent (ANSYS Inc., Canonsburg, PA, USA, R2022) was used to solve the set of fluid equations using the finite-volume scheme. The flow and pressure fields in the lumen were calculated by numerically solving the equations governing continuity and momentum in the steady-state fluid domain:

∇⋅V=0

$$\rho \left(V\cdot \nabla \right)V=-\nabla p+\mu \nabla^{2}V$$

where *p* is static pressure, **V** is the velocity vector, and ρ and μ are the density and dynamic viscosity of the blood, respectively. The blood was assumed to be homogenous, incompressible (ρ = 1 gr/mL), and Newtonian (with viscosity μ = 3.5 cP). The model’s geometry was similar to the experimental model. The numerical model was previously used and validated [50,51]. The numerical mesh (shown in Figure 5) consisted of 2,608,560 tetrahedral elements. The SST k-ω low-Reynolds numerical model was used [52].

The analysis included 12 different simulations of the fluid domain of the aortic arch with inlet flow conditions similar to the experiments. The boundary conditions were set as the inlet axial velocity (*u =* 0.21–0.53 m/s) combined with a perpendicular angular velocity of ω = 0 rad/s for the jet inlet, ω = 900 to 2000 rad/s for the CW case, and ω = (−900 to −2000) rad/s for the CCW case (as listed in Table 1). Stress-free outlet conditions were imposed at the descending aorta outlet.

## 3. Results

**Figure 6:**
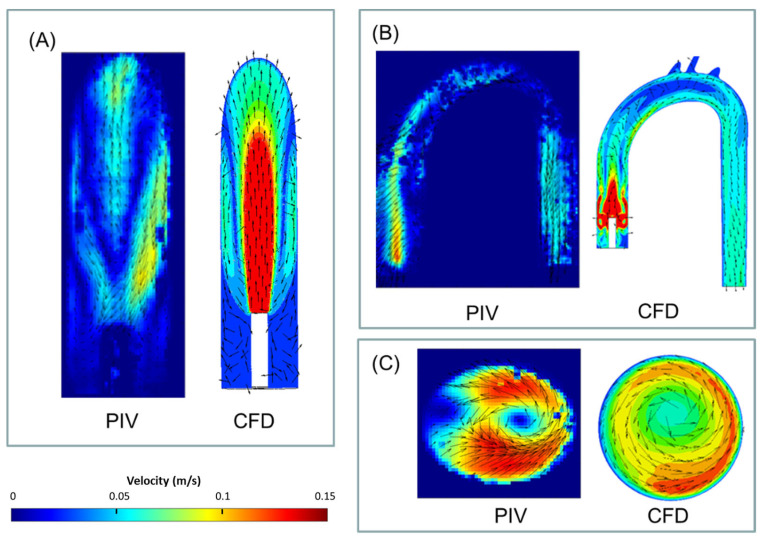
Examples of velocity (vectors and magnitudes) comparison between the obtained PIV and CFD results in three representative cross-sections and cases (2.5 L/min): (**A**) jet case, sagittal section, (**B**) CW case, coronal section, and (**C**) CCW case, distal section. Note that for the numerical results, velocities above 0.15 m/s are colored in red.

Figure 6 presents examples of the velocity vectors as obtained from the PIV and CFD analyses for the three representative cross-sections (purple, orange, and blue) and cases (jet, CW, and CCW). For comparison, the 3D CFD results are projected on similar sections as examined in the PIV analysis and can be quantitatively compared to the 2D PIV velocity maps. The complete set of results obtained from the PIV and the CFD are detailed in the Supplementary Materials. The comparison between the two methods shows that while the PIV failed to capture some of the high-velocity vectors, the main flow characteristics were preserved.

**Figure 7:**
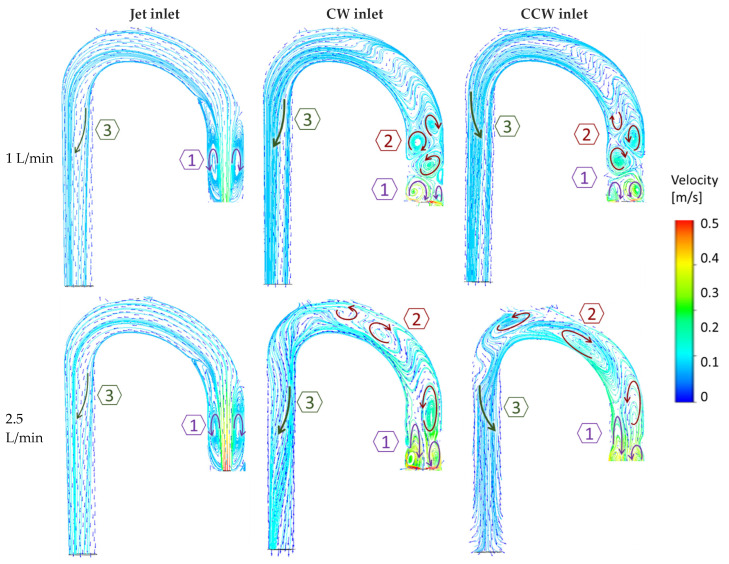
Velocity vectors and streamlines with two example flow rates (1 and 2.5 L/min) in the three flow configurations (jet, CW, and CCW), delineate three dominant phenomena in the flow field. Arrows indicate vortices’ directions.

**Figure 8:**
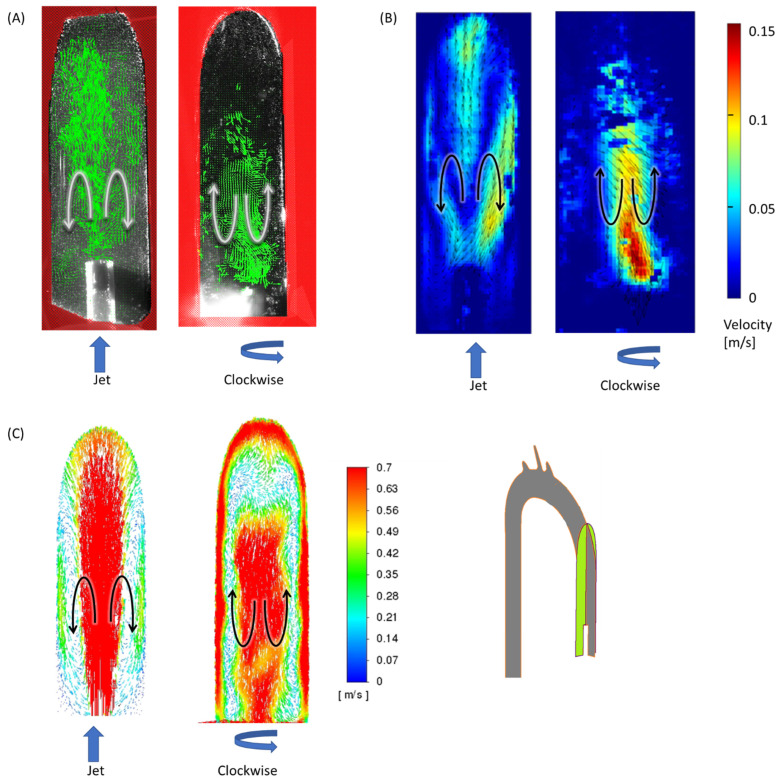
Reverse flow of the incoming jet (phenomenon no. 1) in the sagittal section at 2.5 L/min for the jet inlet (left) and CW inlet (right): (**A**) results of PIV vectors, (**B**) velocities, and (**C**) CFD velocity vectors. Black/white arrows indicate flow directions and blue arrows indicate the impeller’s rotation.

The examined cases exhibited three key flow characteristics along the aorta (identified by number in Figure 7), which may help to highlight the main differences among the cases. These phenomena are integrated into the global flow, and they may have an impact on one another. The first phenomenon (1) is the *reverse flow* as a result of the incoming jet. In the case of the jet inlet, an upward vortex ring formed at the entrance. In contrast, in the cases with a swirling inlet, the reverse flow was inverted, and a downward flow was observed from the center toward the circumferences, as shown in Figure 8.

The second phenomenon (2) is characterized by multiple *local alternating vortices* shed from the rotating impeller. These vortices were not observed in the jet inlet case. At high flow rates, these shed vortices were washed downstream toward the arch.

**Figure 9:**
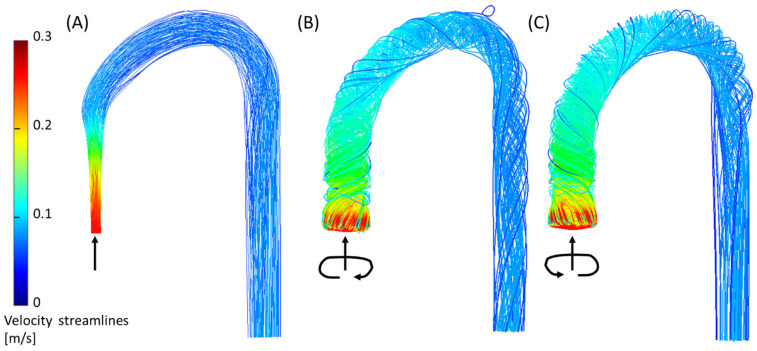
Helical flow in the descending aorta (phenomenon no. 3) from the CFD simulation for the three inlet cases (2.5 L/min): (**A**) jet inlet, (**B**) CW inlet, and (**C**) CCW inlet. Arrows indicate the directions of the inlet flow.

The third feature (3) is the *helical flow* in the descending aorta. In the jet case, a minor CW helical flow was observed at the arch, which faded along the descending aorta (Figure 9A). In the CW case (Figure 9B), the CW helical flow was stronger than the jet case and was kept along the descending aorta. In the CCW (Figure 9C), the helical flow in the arch was in the opposite direction of the natural flow (CW), and it faded along the descending aorta.

**Figure 10:**
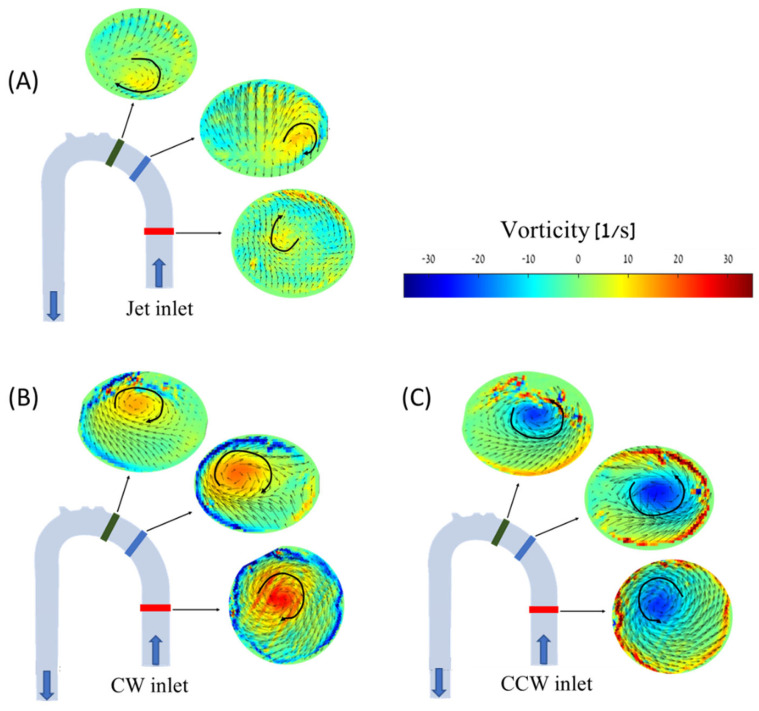
PIV vorticity maps for the different cases and cross-sections along the aorta model at 2.5 L/min: (**A**) jet inlet, (**B**) CW inlet, (**C**) CCW inlet. Blue arrows indicate flow direction, and black arrows indicate vorticities’ directions.

Figure 10 shows the development of the transverse vortical flow along the ascending aorta in the three cases at a flow rate of 2.5 L/min. In the case of the non-swirling jet inlet (Figure 10A), two opposing vortices appeared in the proximal (red) section, representing the upward vortex ring in this region. In the next (middle and distal) sections, the clockwise vortex became stronger and dominant in the helical flow in the descending aorta.

**Figure 11:**
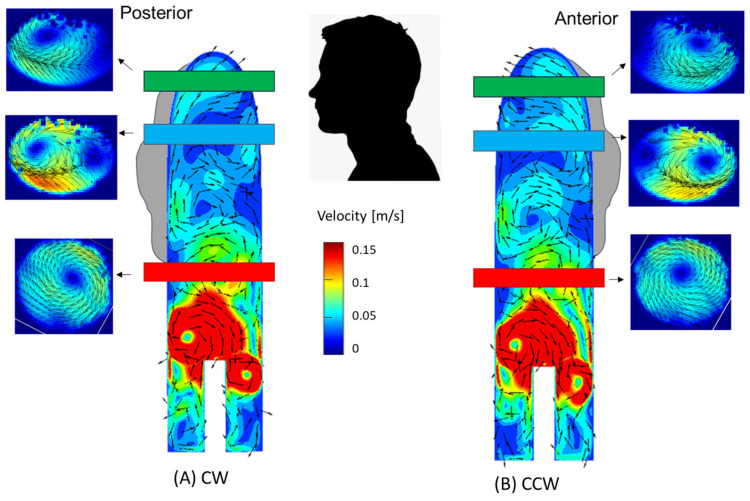
Locations of the vortices in the aorta—a combination of velocity (vectors and magnitudes) in the transverse cross-sections (PIV) and sagittal sections (CFD). (**A**) CW inlet case, (**B**) CCW inlet case. Note that for the numerical results, velocities above 0.15 m/s are colored in red.

In the cases of the CW inlet and the CCW inlet, a similar structure was observed, but in different directions and locations. In the CW inlet case (Figure 10B), a central CW vortex was seen in the proximal section. In the middle and distal sections, this vortex weakened and traveled toward the posterior wall of the aorta (as shown in Figure 11). In the CCW case (Figure 10C), a similar central vortex was found in the proximal section, but in the opposite direction. In this case, the vortex in the middle and distal sections moved toward the anterior wall while it became weaker.

**Figure 12:**
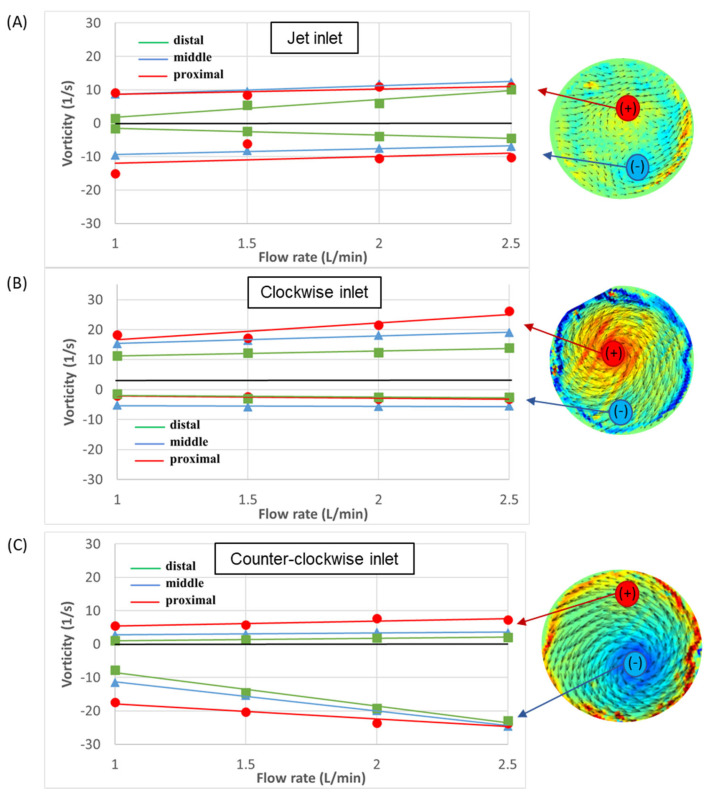
Velocity vectors and vorticity magnitudes as a function of flow rate in the three sections: (**A**) jet inlet, (**B**) CW inlet, and (**C**) CCW inlet. Positive values indicate CW rotational flow and negative values indicate CCW rotational flow.

To further explore the development of vorticity in the aorta, the maximal positive and negative values of vorticity were measured in each cross-section. Positive values indicate CW rotational flow and negative values indicate CCW rotational flow. High positive or negative values indicate strong vortical flow. The magnitudes of the vorticity as a function of the flow rate in the proximal, middle, and distal sections are shown in Figure 12. Overall, high vorticity magnitudes for most flow types and cases were measured in the proximal section. Similarly, the distal section exhibited low positive and negative vorticity magnitudes, implying weaker vortices in the distal location in all the cases.

In addition, in most cases, the positive and negative vorticity magnitudes increased with the flow rates. In the case of the jet inlet, the negative and positive values were of similar magnitudes, while in the CW case, the positive magnitudes were higher, and in the CCW case, the negative magnitudes were higher.

**Figure 13:**
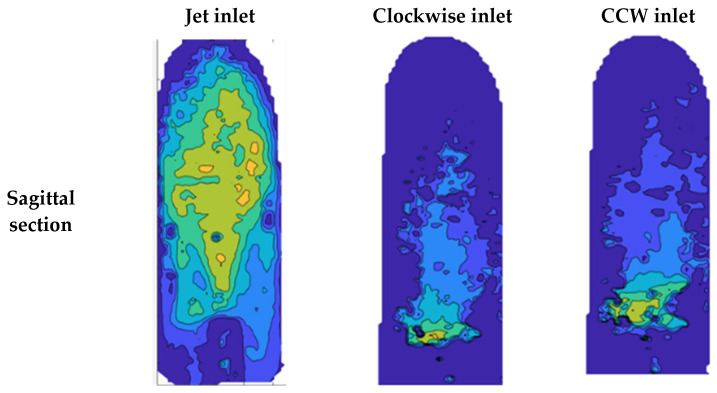

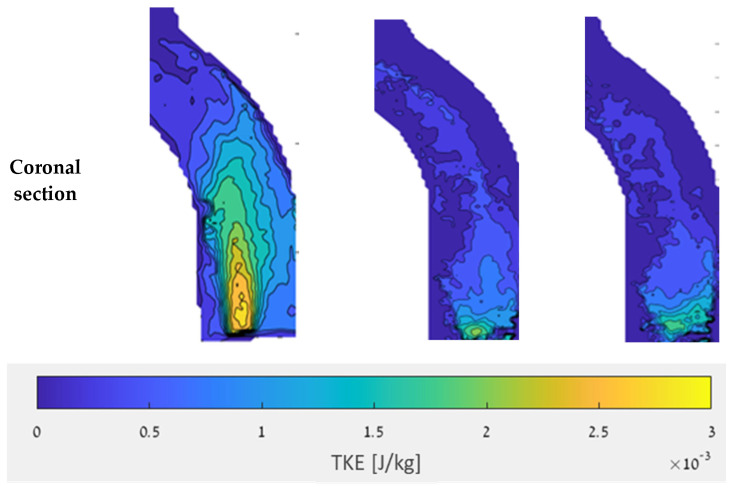
TKE in the sagittal and coronal sections with a flow rate of 2.5 L/min.

Figure 13 shows examples of local TKE distribution maps for the different examined cases at 2.5 L/min in the sagittal and coronal sections (for detailed maps of the TKE results of all sections and flow rates see the Supplementary Materials). In the coronal section, the focus was on the entrance to the aorta and the ascending aorta. High TKE values (up to 0.003 J/kg) were widely scattered in the jet inlet case. Both the CW and CCW inlets showed significantly smaller distributions of high TKE values compared to the jet inlet.

**Figure 14:**
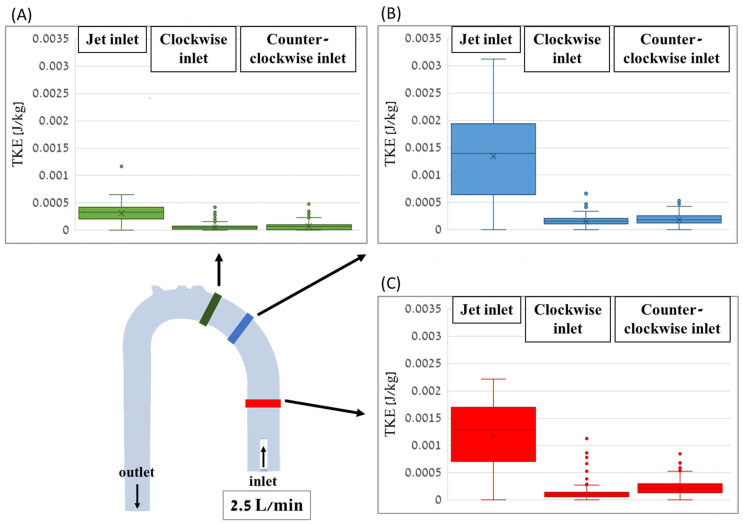
Box plots of the TKE distribution values for the different cases along the distal (**A**), middle (**B**), and proximal (**C**) cross-sections of the aorta with a flow rate of 2.5 L/min.

Comparisons of the TKE value distributions for the different cases are shown in box plots in Figure 14 for the three sections along the aorta with a flow rate of 2.5 L/min. In all sections, the jet inlet case had the highest TKE values, while the CW inlet and CCW inlet cases had significantly lower TKE values.

## 4. Discussion

This study examined the flow in the aorta in the presence of an MCS device using combined experimental (PIV) and numerical (CFD) analyses. It was shown that the flow emerging from discharge MCS pumps with a non-swirling jet inlet [6] combined well with the natural flow, inducing a moderate CW helical flow in the aortic arch and descending aorta. However, it may induce a stronger direct jet with a relatively high TKE, which might promote structural pathologies of the vessel and release emboli from a calcified aorta wall [53,54].

On the other hand, the flow induced by suction-pump configurations with distal rotating impellers was dominated by an inverse vortex ring with a vortical flow near the aortic valve and in the ascending aorta. These vortices decayed on their way downstream, thus spreading the emerging jet and resulting in the decay of TKE in the ascending aorta. However, the local vortices formed near the aortic wall induced local disturbances. CW rotation tended to draw the vortices toward the posterior wall of the aorta, while CCW rotation did so toward the anterior wall. Such proximity of the vortex to one of the anterior or posterior walls of the aorta during long-term treatment might promote local pathologies, such as aortic aneurysm, dissection, and coarctation [28].

In both swirling jet cases, high flow rates washed out the local vortices near the pump inlet downstream toward the aortic arch and distributed them along the ascending aorta. While CW swirl combines better with the natural CW helical flow of the aorta formed by the curvature of the aorta [19], a suction impeller that rotates in the CCW direction [32] induces a non-physiological helical flow in the descending aorta, which may also affect blood flow to the branching vessels (to the upper body and renal arteries) [17] and should be further studied. Therefore, if a suction pump is considered, it could be better to have a CW swirling flow to better combine with the natural physiological helical flow.

The main conclusions are as follows:

- Swirling flow reduces the TKE of the pump’s jet inlet and improves the flow distribution near the aortic valve and the ascending aorta (and may reduce the risk of release of emboli from the aorta wall);
- Swirling flow increases the dominance of vortical flow near the valve and in the ascending aorta;
- In the case of the CW inlet case, the vortex was drawn toward the posterior wall of the aorta, while in the CCW inlet case, the vortex was drawn toward the anterior wall of the aorta;
- A high flow rate washed out the local turbulence near the pump inlet downstream toward the aortic arch, and thus, the local vortices formed near the outlet were more distributed in the ascending aorta;
- pLVAD with a CCW rotating impeller (such as in the Impella pump) induced non-physiological CCW helical flow in the descending aorta (which is the opposite of the natural helical flow);
- Clockwise swirl combined better with the natural helical flow.

The study has some limitations. The PIV method’s primary limitation is its inability to capture global 3D flow, and it is particularly inaccurate at high velocities (above 0.2 m/s). Specifically, the proximal (red) section, which was far from the camera and hard to capture, provided lower-quality images. Therefore, the PIV results obtained from the proximal section should be interpreted with caution. Although the CFD results provide some complementary outlook of the 3D flow, and the TKE results presented for these sections (in the range of 0–0.003 J/kg) are in good agreement with the values reported in the literature (e.g., [29], the estimation of TKE should be considered mainly qualitatively.

Moreover, capturing the flow in cross-sections perpendicular to the main flow (proximal, middle, and distal sections) was challenging because of the narrow width of the laser sheet, in which the particles lingered for a very short time (and thus, might have escaped the light sheet before the next frame). To solve this, we used a lens that provided a wider laser sheet (2 mm). By using a wider light sheet, the high-speed camera could better capture the particles while they traveled across the cross-section.

In the experiments, we used two pumps in a row. The DC pump, placed in the reservoir, provided the forward flow rate in the “jet inlet” case (simulating a “discharge pump”). In the CW and CCW cases (simulating a “suction pump”), the rotating impeller placed in the inlet of the aorta provided both the forward and swirling components of the flow. However, due to the impeller design, some differences in the flow rate were measured between the CW and CCW configurations. Therefore, the auxiliary pump and the valve were also used in these cases to regulate the required pump head demand of the system and allow for the flow rates as defined in Table 1. In this way, the combination of the main “swirling” pump with the auxiliary “forward” pump allowed us to examine the cases with the jet inlet, CW, and CCW, all with the same forward flow rates.

Despite the simplifications and limitations listed above, the flow rates measured by the PIV technique agree with the flow rates measured using alternative measurements, and the vorticity and TKE values obtained in this study are in good agreement with those in the literature and obtained through numerical simulation [50]. Therefore, the presented results may identify the key variables affecting hemodynamics in the examined cases.

Future work may include the following:

- Examining the effect of CCW helical flow in the descending aorta on the flow to the branching arteries (e.g., coronaries, subclavian, carotid, and renal);
- Examining the effects of different rotational velocities on the flow;
- Examining the effects of a beating heart and the obtained combined flow (including the aortic valve’s dynamics);
- Examining the effects of the different flow features on each other.

The main implications of this study are related to the design of MCS devices concerning the resulting aortic flow. This study may shed light on future MCS development in the pursuit of reducing MCS complications and better treatment of acute heart failure patients.

## Figures and Tables

**Table 1:** Cases studied.

Experiment		Numerical Boundary Conditions		
Flow Rate	Rotational Speed *	Jet Inlet	CW Inlet	CCW Inlet
1 L/min	9000 RPM	u = 0.21 m/s ω = 0 rad/s	u = 0.21 m/s ω = 900 rad/s CW	u = 0.21 m/s ω = 900 rad/s CCW
1.5 L/min	12,000 RPM	u = 0.32 m/s ω = 0 rad/s	u = 0.32 m/s ω = 1270 rad/s CW	u = 0.32 m/s ω = 1270 rad/s CCW
2 L/min	15,500 RPM	u = 0.42 m/s ω = 0 rad/s	u = 0.42 m/s ω = 1630 rad/s CW	u = 0.42 m/s ω = 1630 rad/s CCW
2.5 L/min	19,000 RPM	u = 0.53 m/s ω = 0 rad/s	u = 0.53 m/s ω = 2000 rad/s CW	u = 0.53 m/s ω = 2000 rad/s CCW

* Impeller speed (in RPM) in the experiments for the CW and CCW cases.

## Data Availability

All the relevant data is available in the manuscript or in the Supplementary Materials.

## Supplementary Materials

The following supporting information can be downloaded at: https://www.mdpi.com/article/10.3390/bioengineering11030238/s1. The complete set of results obtained from the PIV and the CFD are detailed in the Supplementary Materials. Table S1: Contour scale for Velocity, Vorticity, and Turbulence kinetic energy. Table S2: Velocity CFD—Jet flow. Table S3: Velocity CFD—Clockwise flow. Table S4: Velocity CFD—Counterclockwise flow. Table S5: Velocity PIV—Jet flow. Table S6: Velocity PIV—Clockwise flow. Table S7: Velocity PIV—Counterclockwise flow. Table S8: Vorticity PIV—Jet flow. Table S9: Vorticity PIV—Clockwise flow. Table S10: Vorticity PIV—Counterclockwise flow. Table S11: TKE PIV—Jet flow. Table S12: TKE PIV—Clockwise flow. Table S13: TKE PIV—Counterclockwise flow.
