# Supplementary material for: The Effect of Mechanical Circulatory Support on Blood Flow in the Ascending Aorta: A Combined Experimental and Computational Study
Source: Bioengineering (Basel). 2024 Feb 28;11(3):238. doi: 10.3390/bioengineering11030238 (PMC10968640; doi:10.3390/bioengineering11030238)
Supplement: Supplementary file 1 [file bioengineering-11-00238-s001.zip › bioengineering-2849507-supplementary.pdf]

# The Effect of Mechanical Circulatory Support on Blood Flow in the Ascending Aorta: A Combined Experimental and Computational Study

**Sapir Hazan Shenberger and Idit Avrahami**

# Supplemental material

## 1. Global Legends and Contour scale

**Table S1:** Contour scale **for Velocity, Vorticity, and Turbulence kinetic energy.**

| | Proximal, middle and distal sections | Coronal and sagittal sections |
|---|---|---|
| Velocity | 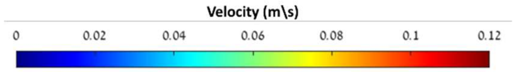  | 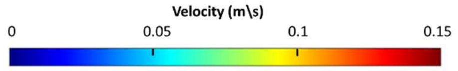  |
| Vorticity | 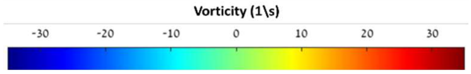  | 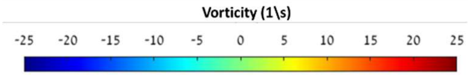  |
| Turbulence kinetic energy | 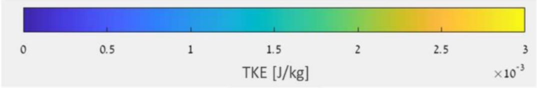  | |

2. **Velocity vectors and magnitudes, CFD**

**Table S2: Velocity CFD- Jet flow**

| | **1 L/min** | **1.5 L/min** | **2 L/min** | **2.5 L/min** |
|---|---|---|---|---|
| **Distal section** | 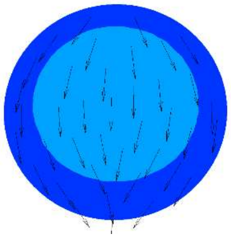 | 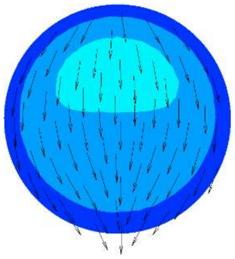 | 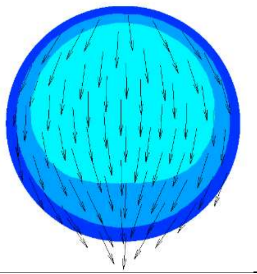 | 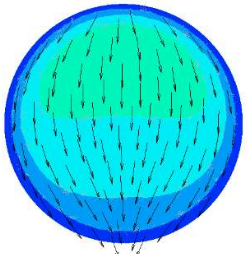 |
| **Middle section** | 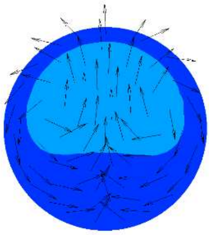 | 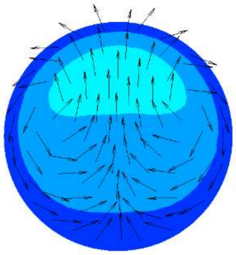 | 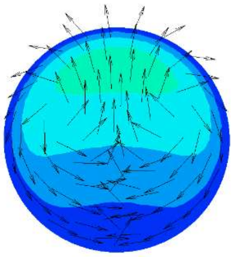 | 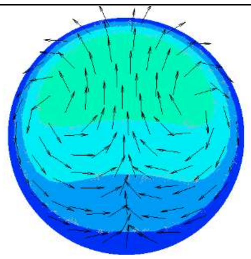 |
| **Proximal section** | 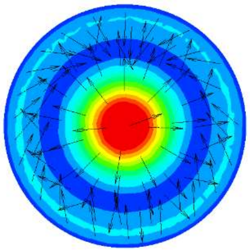 | 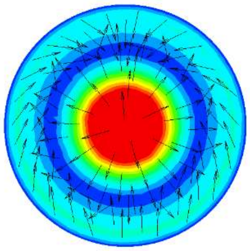 | 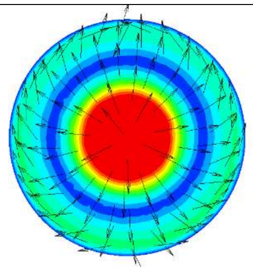 | 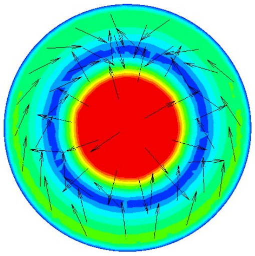 |
| **Sagittal section** | 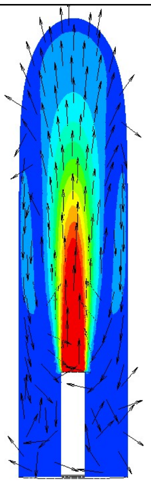 | 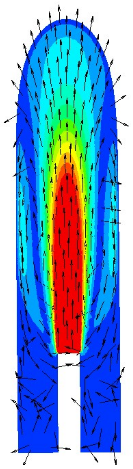 | 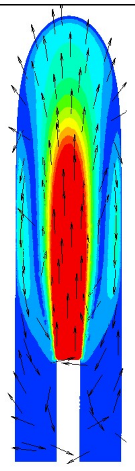 | 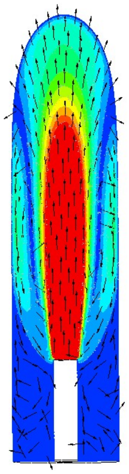 |

| | | | | |
|---|---|---|---|---|
| **Coronal section** | 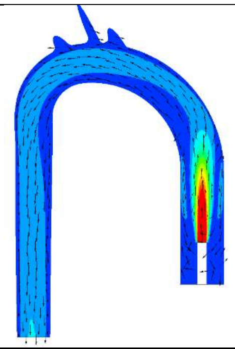 | 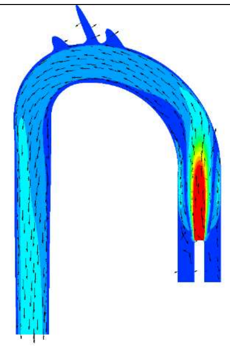 | 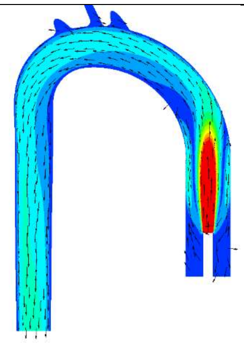 | 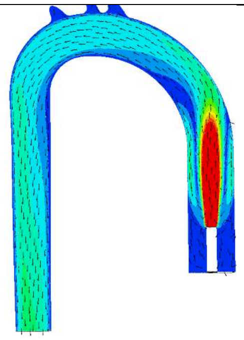 |

**Table S3: Velocity CFD- Clockwise flow**

| | 1 L/min | 1.5 L/min | 2 L/min | 2.5 L/min |
|---|---|---|---|---|
| Distal section | 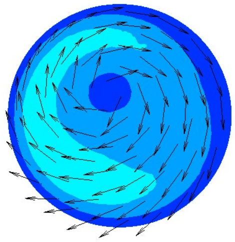 | 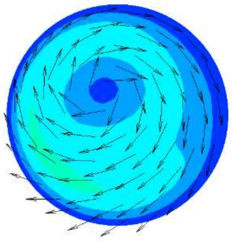 | 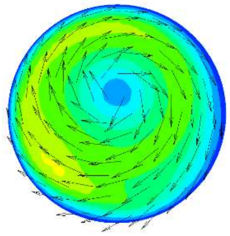 | 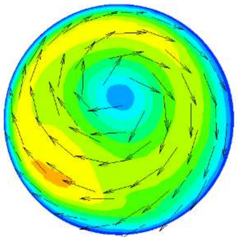 |
| Middle section | 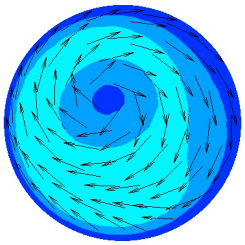 | 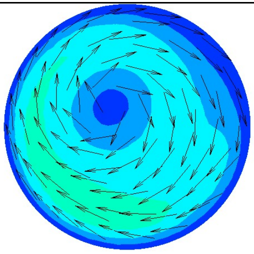 | 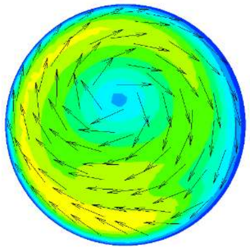 | 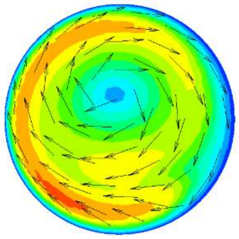 |
| Proximal section | 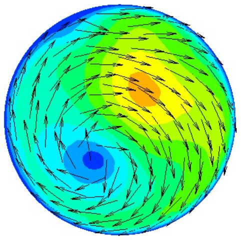 | 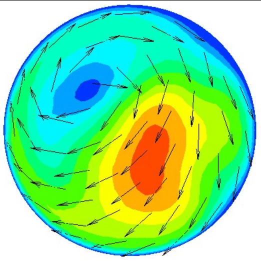 | 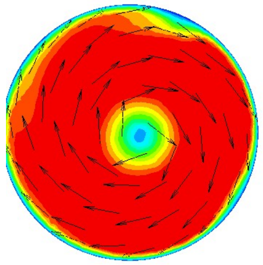 | 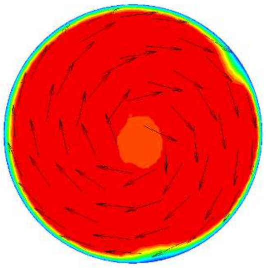 |
| Sagittal section | 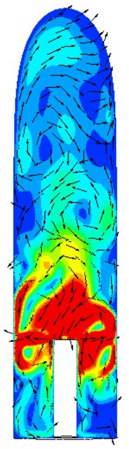 | 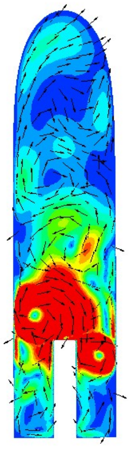 | 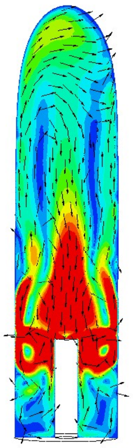 | 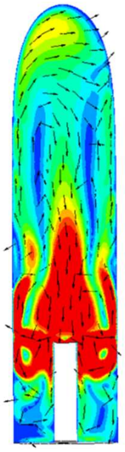 |
| Coronal section | 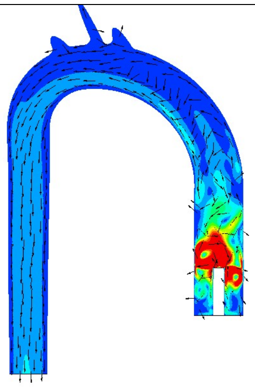 | 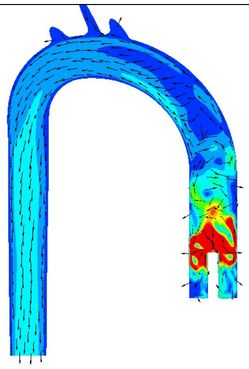 | 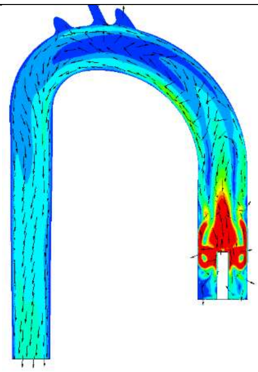 | 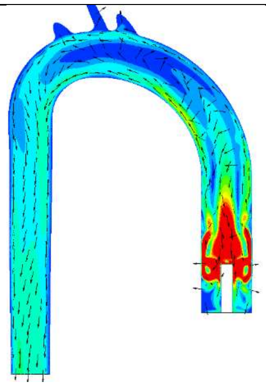 |

**Table S4: Velocity CFD- Counterclockwise flow**

| | 1 L/min | 1.5 L/min | 2 L/min | 2.5 L/min |
|---|---|---|---|---|
| **Distal section** | 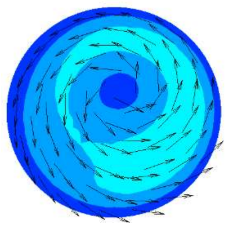 | 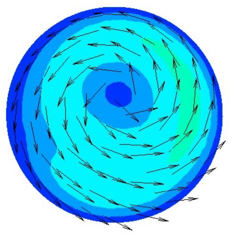 | 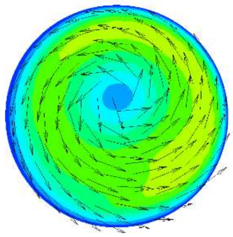 | 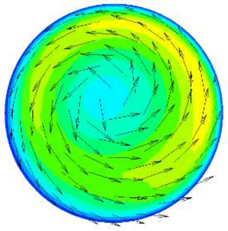 |
| **Middle section** | 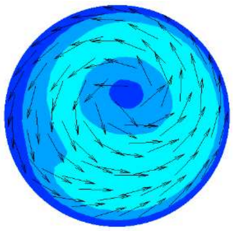 | 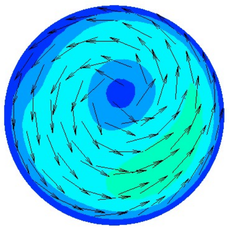 | 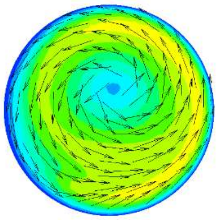 | 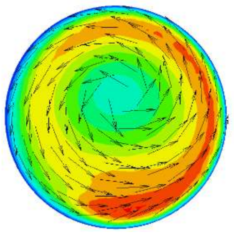 |
| **Proximal section** | 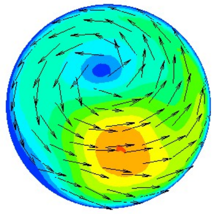 | 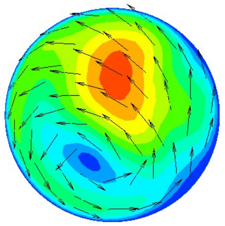 | 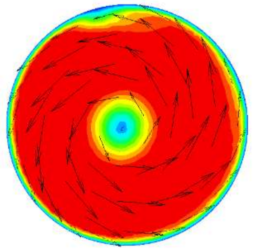 | 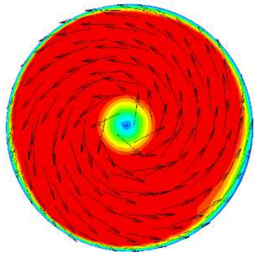 |
| **Sagittal section** | 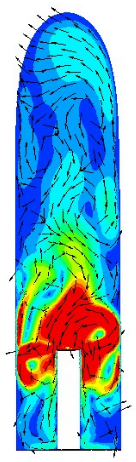 | 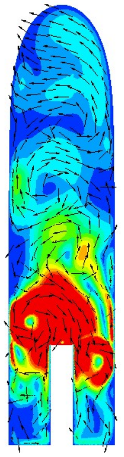 | 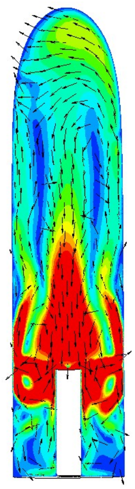 | 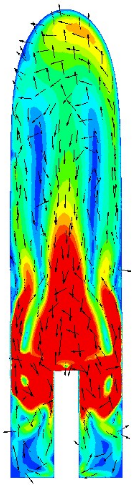 |

| | | | | |
|---|---|---|---|---|
| **Coronal section** | 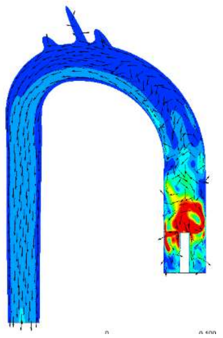 | 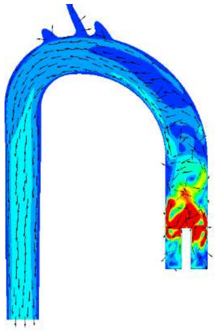 | 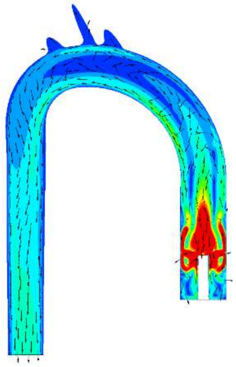 | 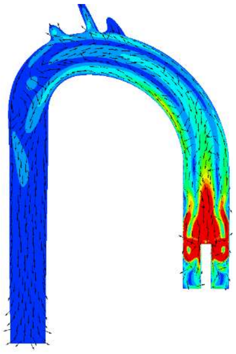 |

3. **Velocity vectors and magnitudes, PIV**

**Table S5: Velocity PIV- Jet flow**

| | 1 L/min | 1.5 L/min | 2 L/min | 2.5 L/min |
|---|---|---|---|---|
| Distal section | 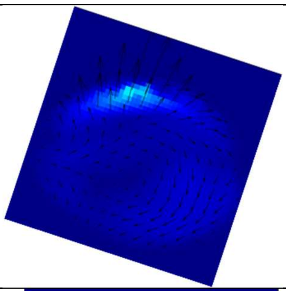 | 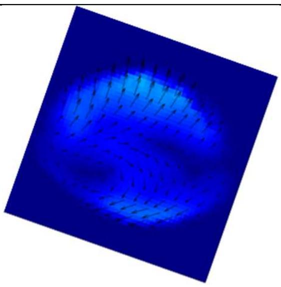 | 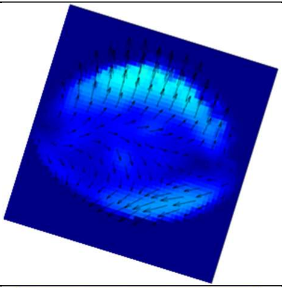 | 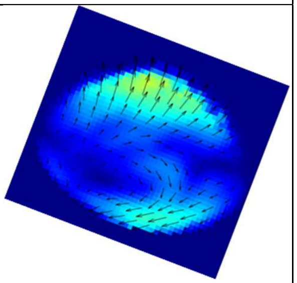 |
| Middle section | 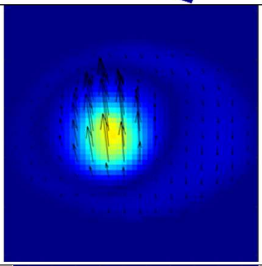 | 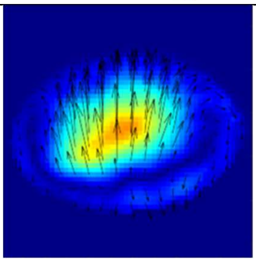 | 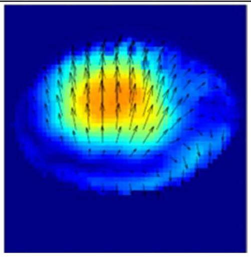 | 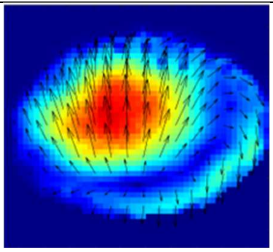 |
| Proximal section | 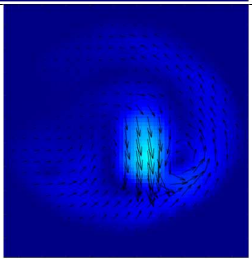 | 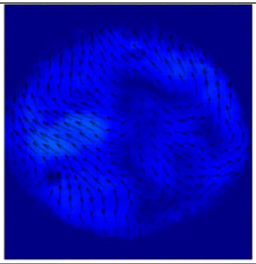 | 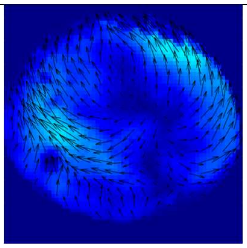 | 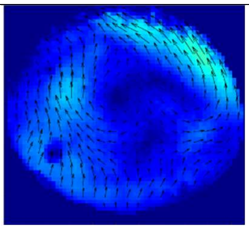 |
| Sagittal section | 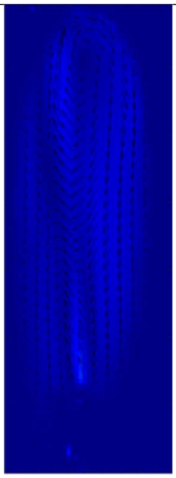 | 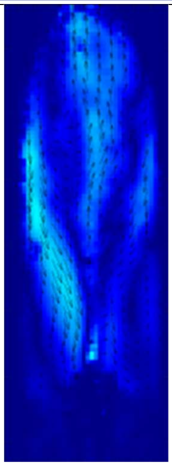 | 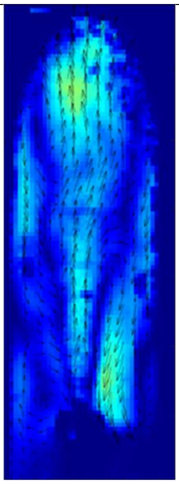 | 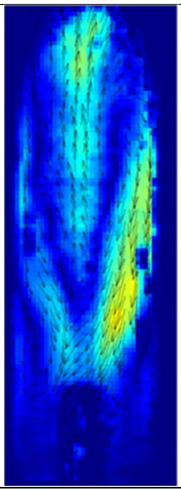 |

| | | | | |
|---|---|---|---|---|
| **Coronal section** | 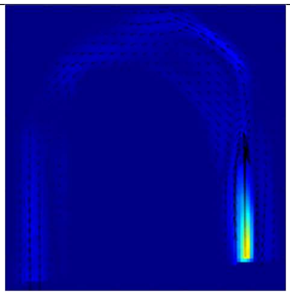 | 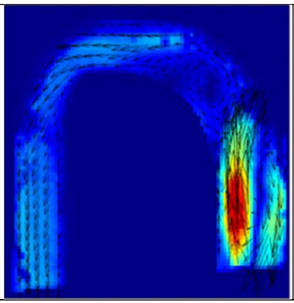 | 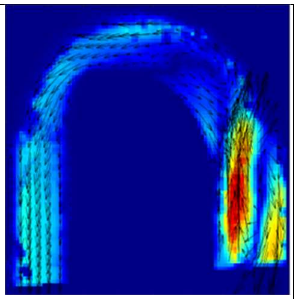 | 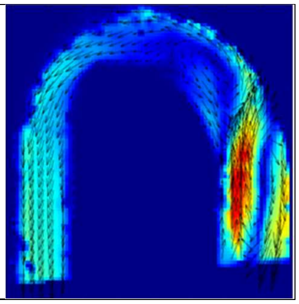 |

**Table S6: Velocity PIV- - Clockwise flow**

| | 1 L/min | 1.5 L/min | 2 L/min | 2.5 L/min |
|---|---|---|---|---|
| Distal section | 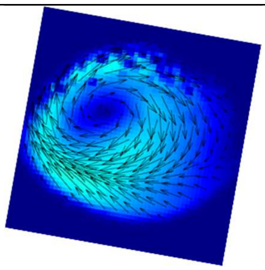 | 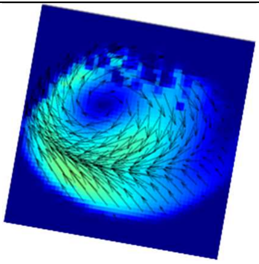 | 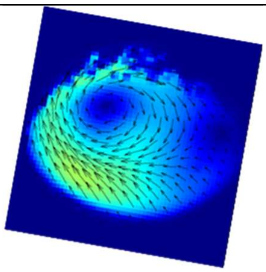 | 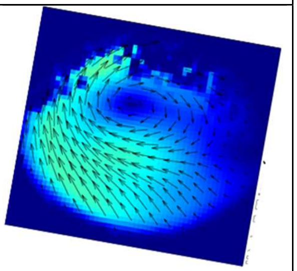 |
| Middle section | 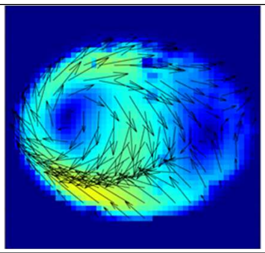 | 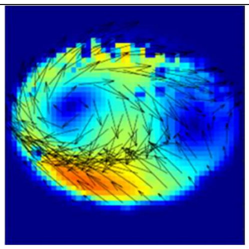 | 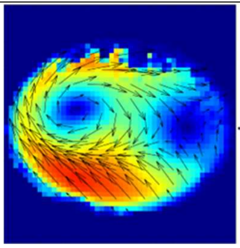 | 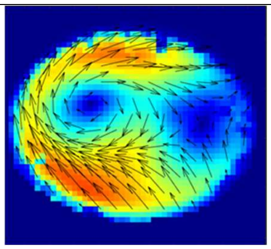 |
| Proximal section | 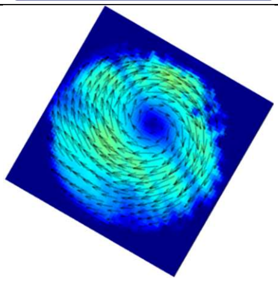 | 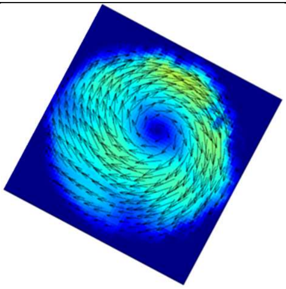 | 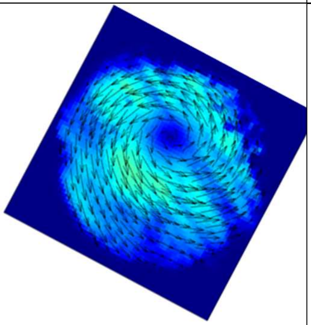 | 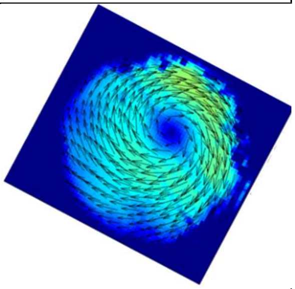 |
| Sagittal section | 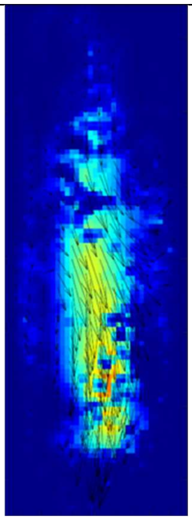 | 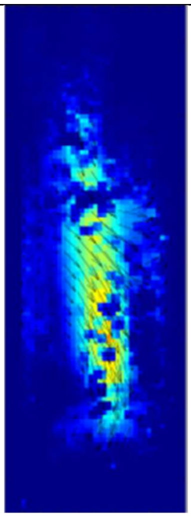 | 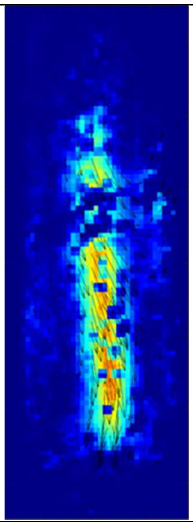 | 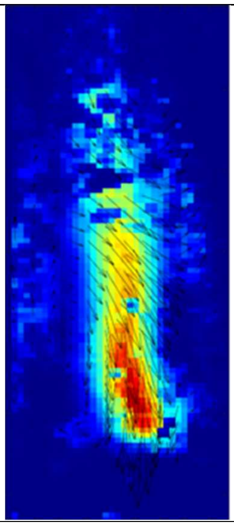 |

| | | | | |
|---|---|---|---|---|
| **Coronal section** | 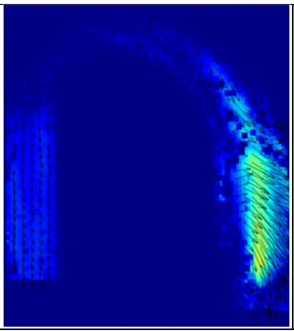 | 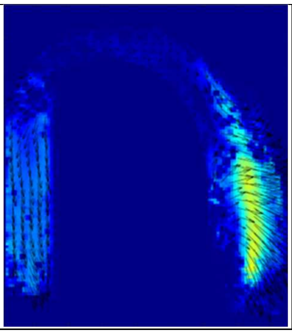 | 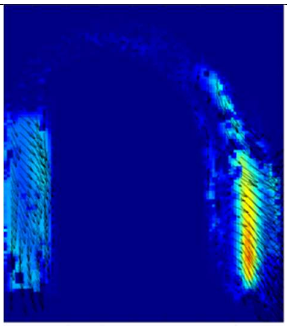 | 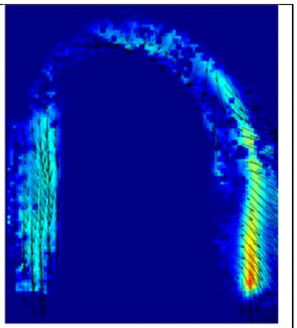 |

Table S7: Velocity PIV- - Counterclockwise flow

| | 1 L/min | 1.5 L/min | 2 L/min | 2.5 L/min |
|---|---|---|---|---|
| Distal section | 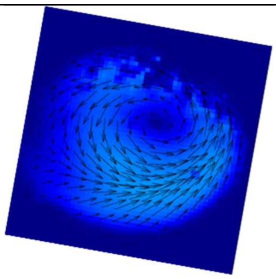 | 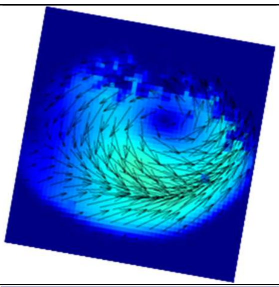 | 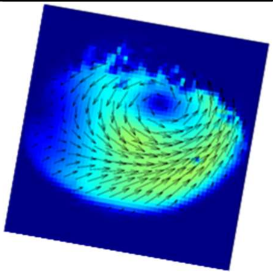 | 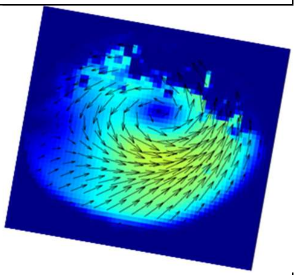 |
| Middle section | 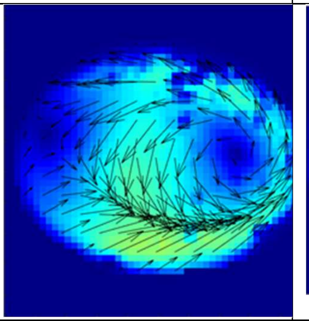 | 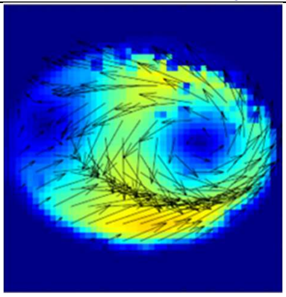 | 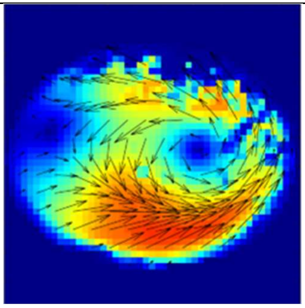 | 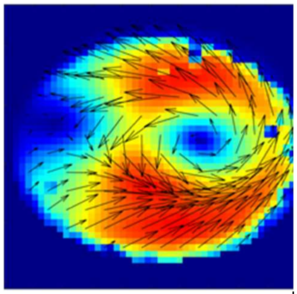 |
| Proximal section | 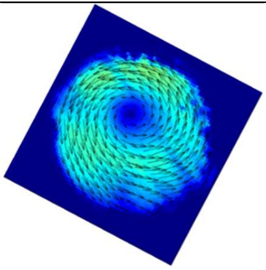 | 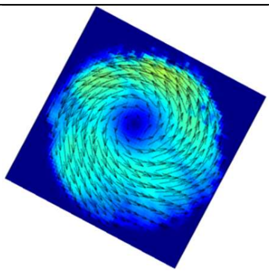 | 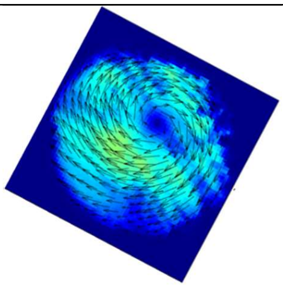 | 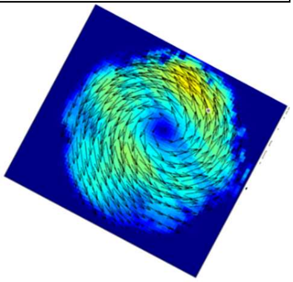 |
| Sagittal section | 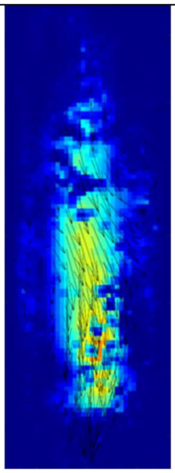 | 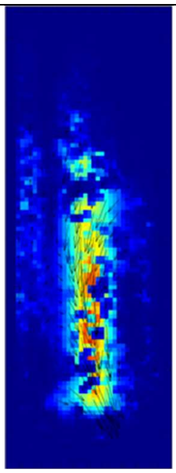 | 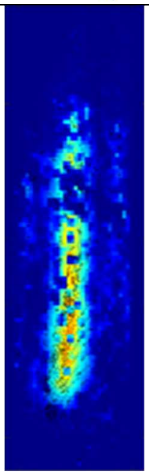 | 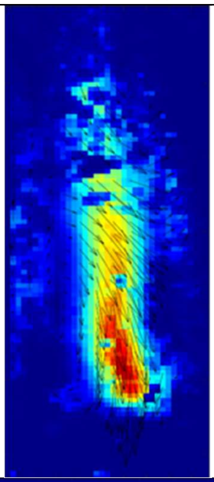 |
| Coronal section | 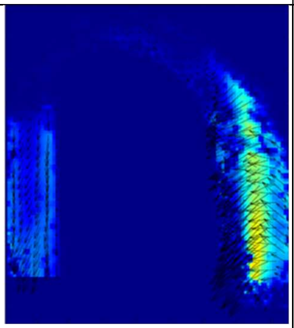 | 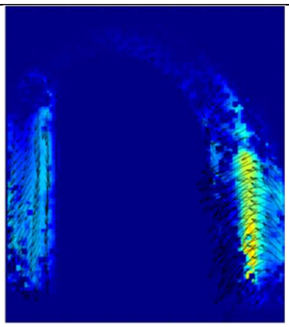 | 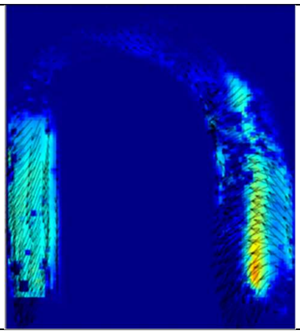 | 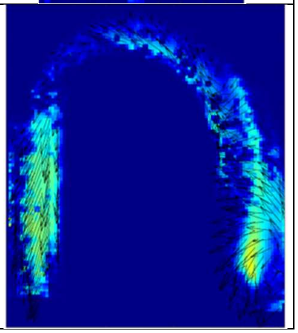 |

## 4. Vorticity vectors and magnitudes, PIV

Table S8: Vorticity PIV -Jet flow

| | 1 L/min | 1.5 L/min | 2 L/min | 2.5 L/min |
|---|---|---|---|---|
| Distal section | 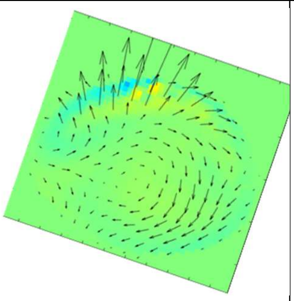 | 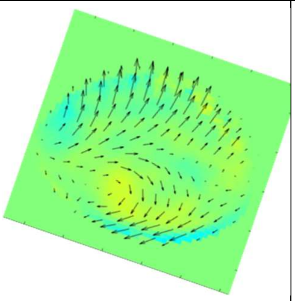 | 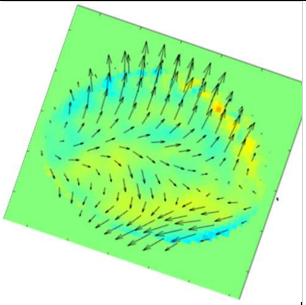 | 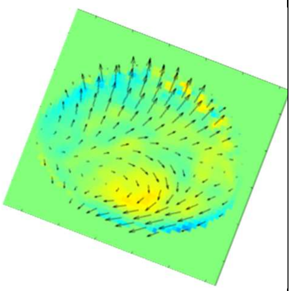 |
| Middle section | 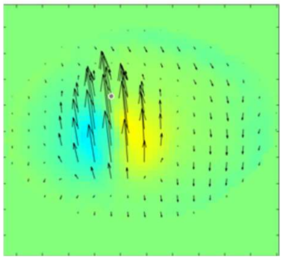 | 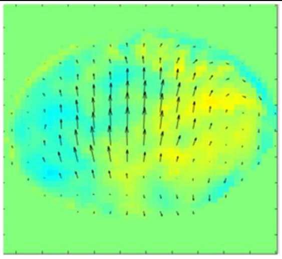 | 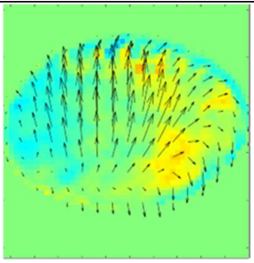 | 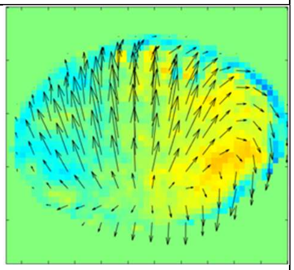 |
| Proximal section | 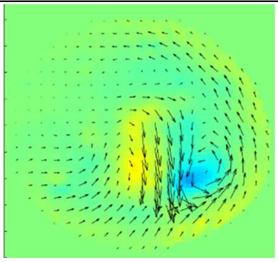 | 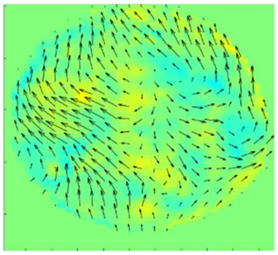 | 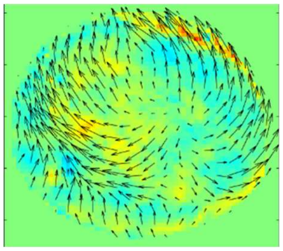 | 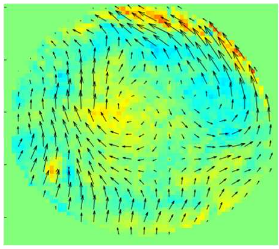 |
| Sagittal section | 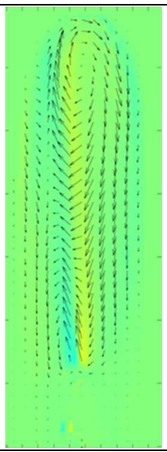 | 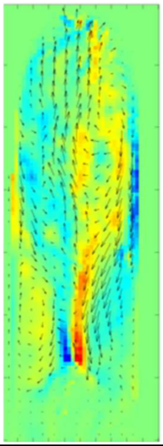 | 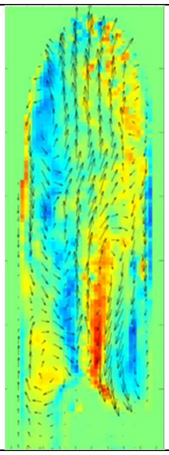 | 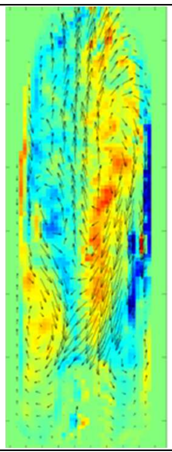 |

**Coronal section**

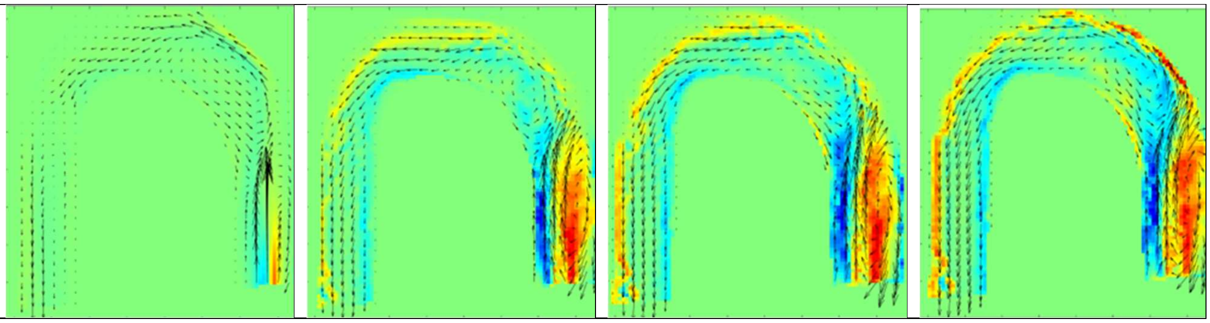

**Table S9: Vorticity PIV -Clockwise flow**

| | 1 L/min | 1.5 L/min | 2 L/min | 2.5 L/min |
|---|---|---|---|---|
| **Distal section** | 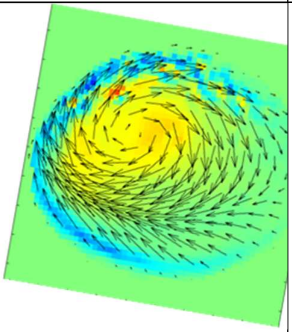 | 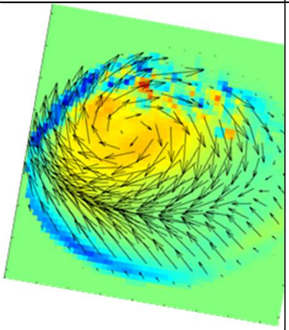 | 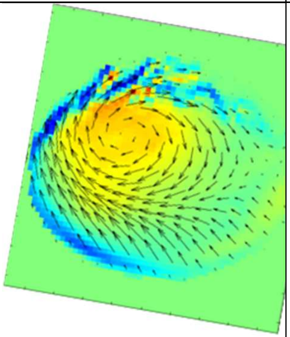 | 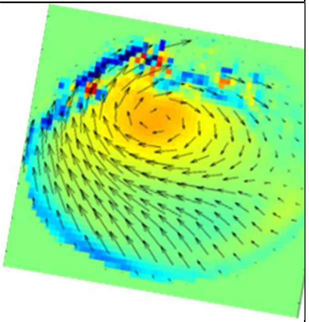 |
| **Middle section** | 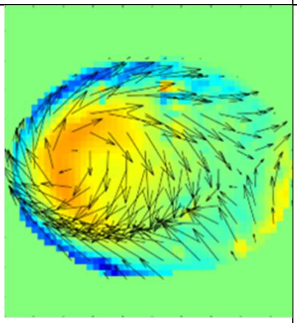 | 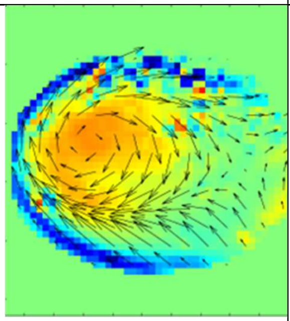 | 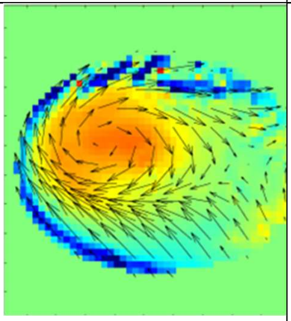 | 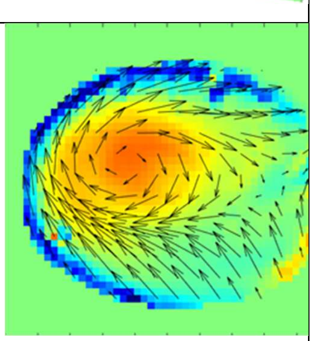 |
| **Proximal section** | 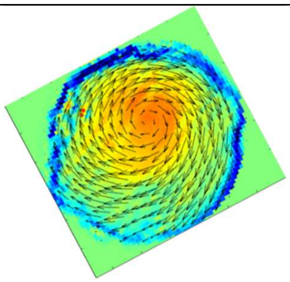 | 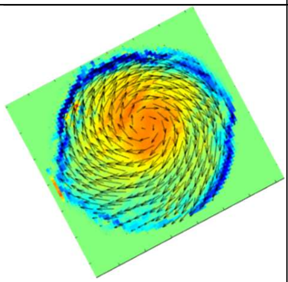 | 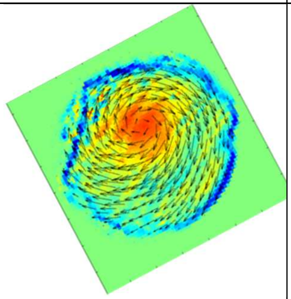 | 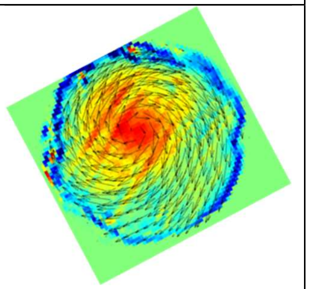 |
| **Sagittal section** | 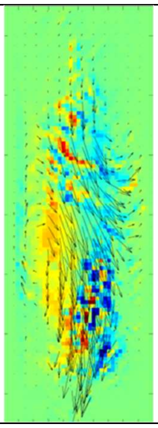 | 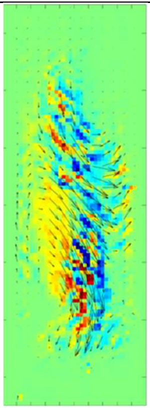 | 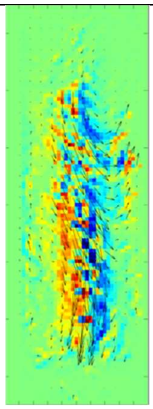 | 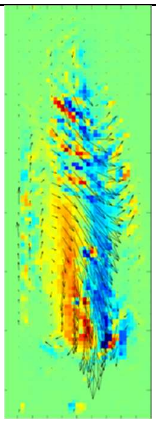 |

| | | | | |
|---|---|---|---|---|
| **Corona l section** | 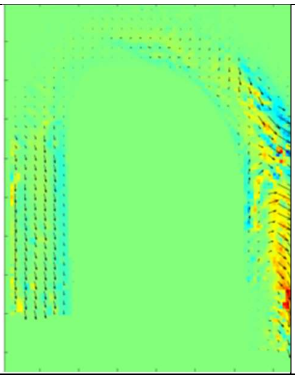 | 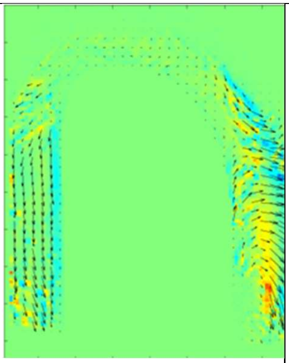 | 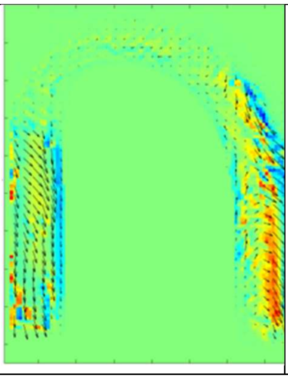 | 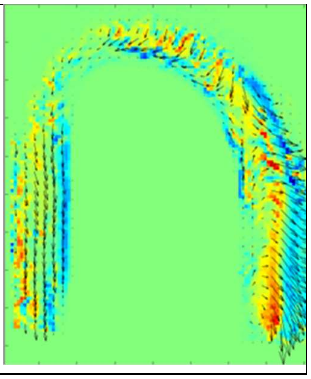 |

**Table S10: Vorticity PIV -Counterclockwise flow**

| | 1 L/min | 1.5 L/min | 2 L/min | 2.5 L/min |
|---|---|---|---|---|
| Distal section | 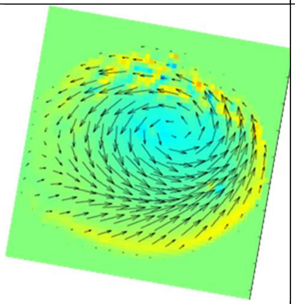 | 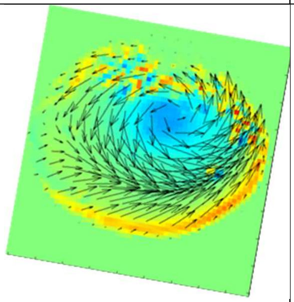 | 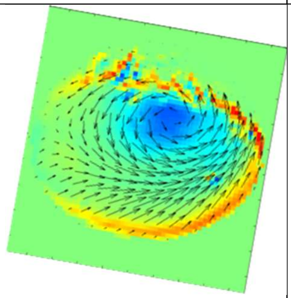 | 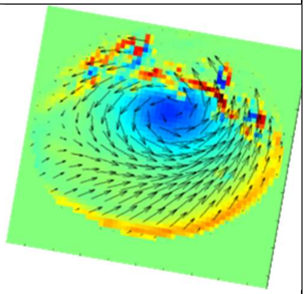 |
| Middle section | 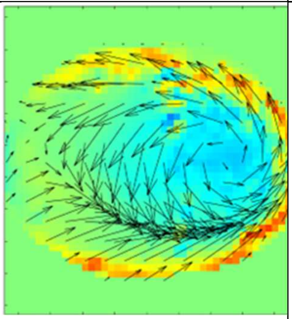 | 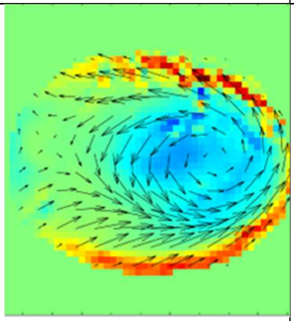 | 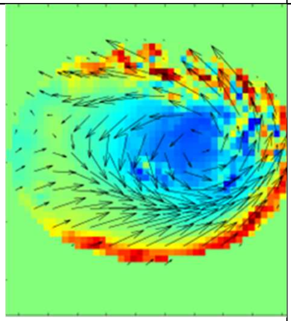 | 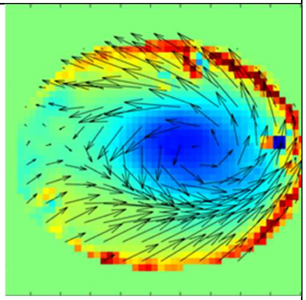 |
| Proxim al section | 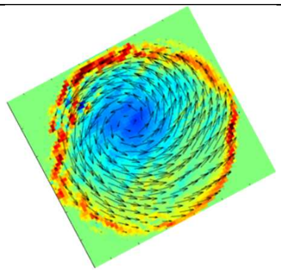 | 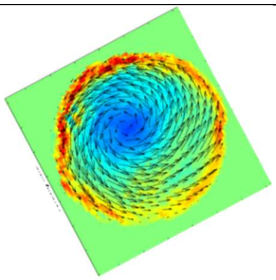 | 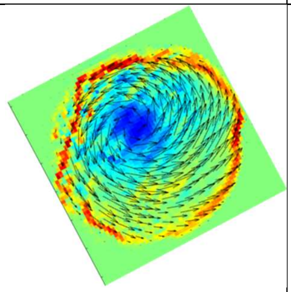 | 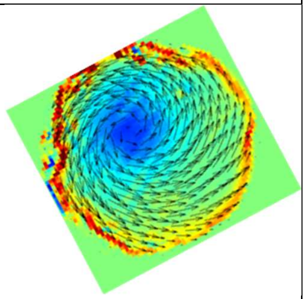 |
| Sagittal section | 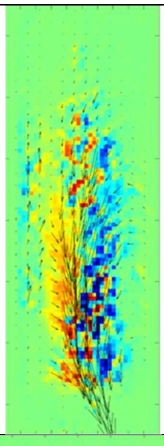 | 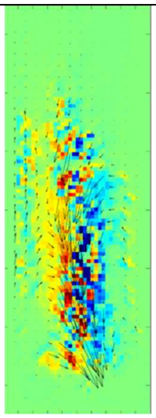 | 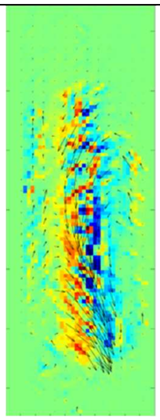 | 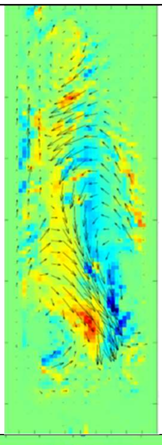 |
| Coronal section | 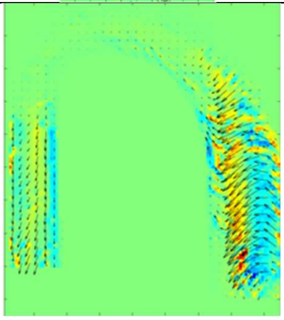 | 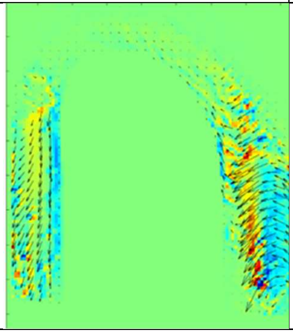 | 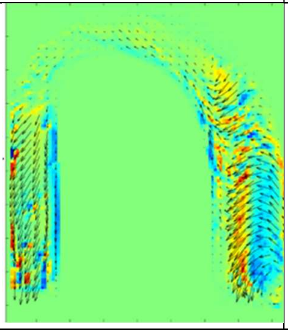 | 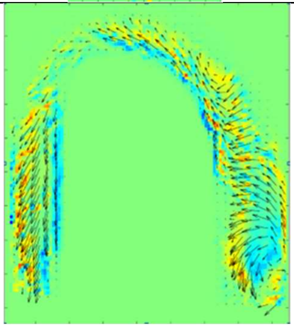 |

5. **TKE, PIV**

**Table S11: TKE PIV -- Jet flow**

| | 1 L/min | 1.5 L/min | 2 L/min | 2.5 L/min |
|---|---|---|---|---|
| Distal section | 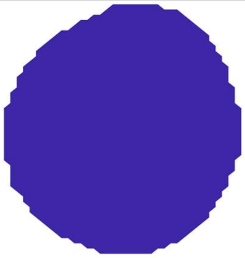 | 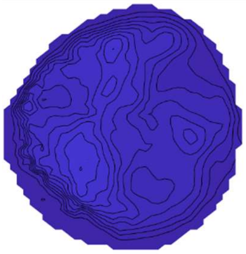 | 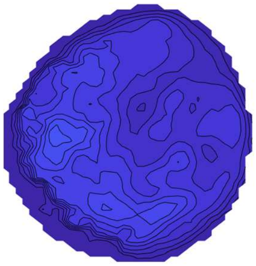 | 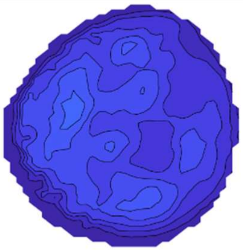 |
| Middle section | 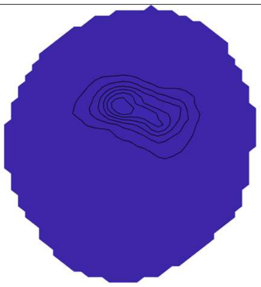 | 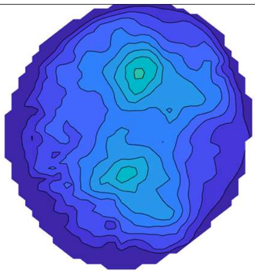 | 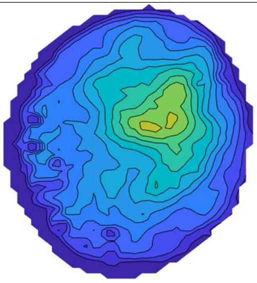 | 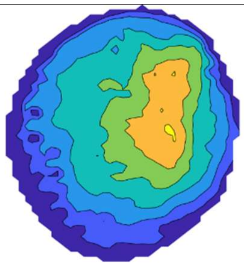 |
| Proximal section | 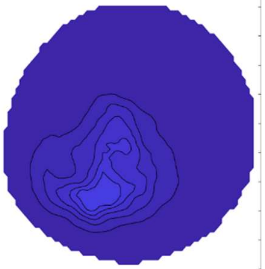 | 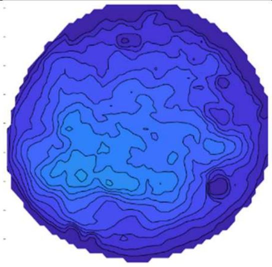 | 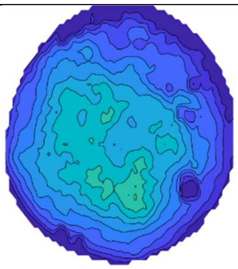 | 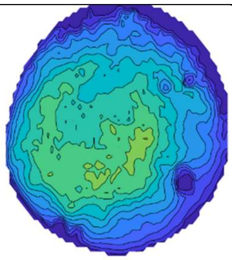 |
| Sagittal section | 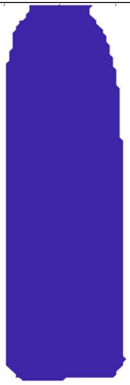 | 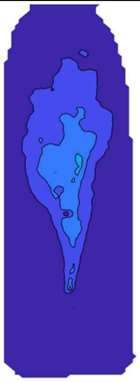 | 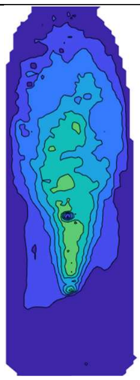 | 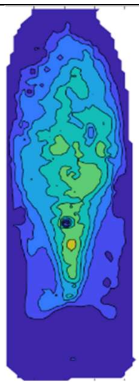 |
| Coronal section | 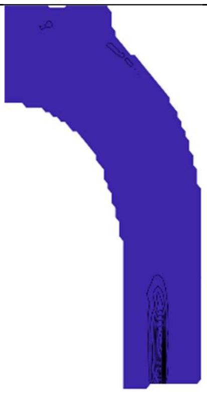 | 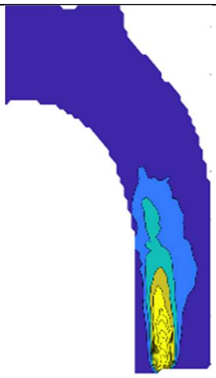 | 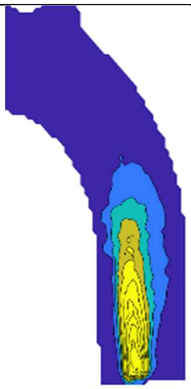 | 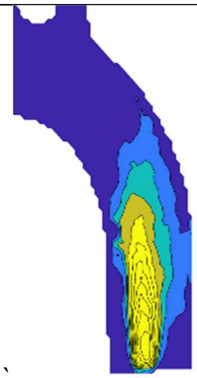 |

Table S12: TKE PIV --Clockwise flow

| | 1 L/min | 1.5 L/min | 2 L/min | 2.5 L/min |
|---|---|---|---|---|
| Distal section | 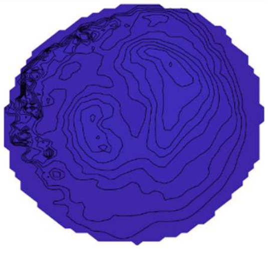 | 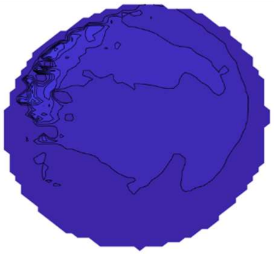 | 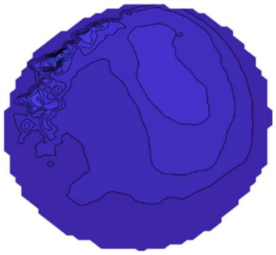 | 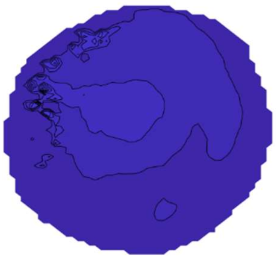 |
| Middle section | 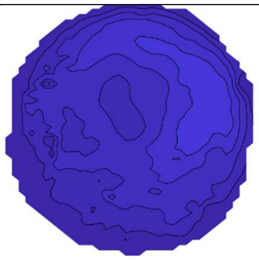 | 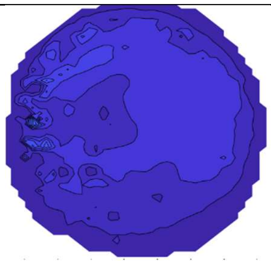 | 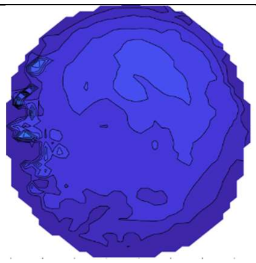 | 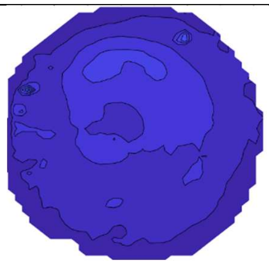 |
| Proximal section | 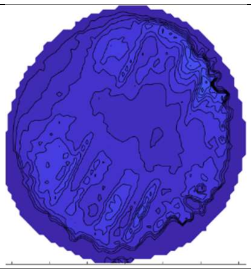 | 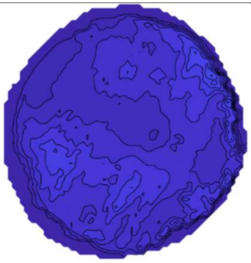 | 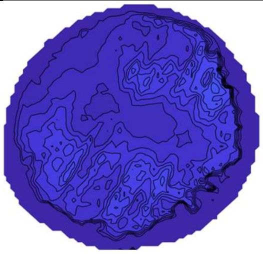 | 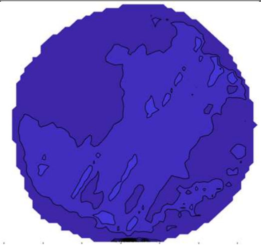 |
| Sagittal section | 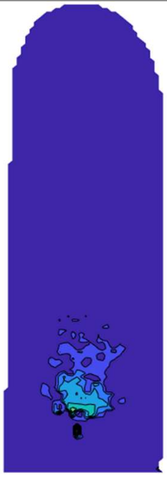 | 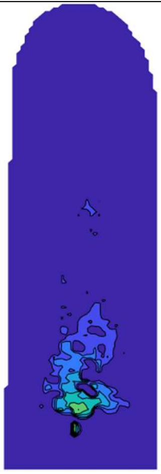 | 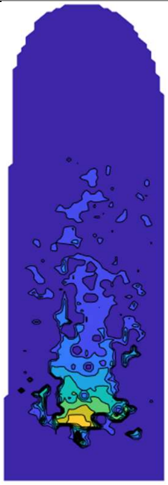 | 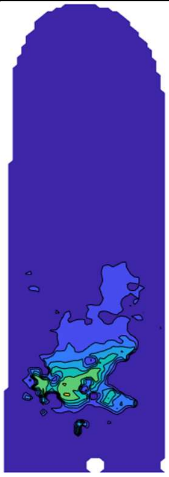 |
| Coronal section | 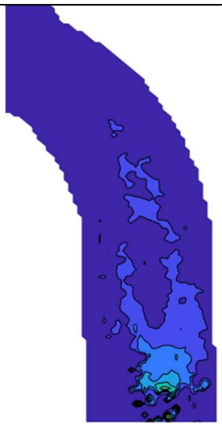 | 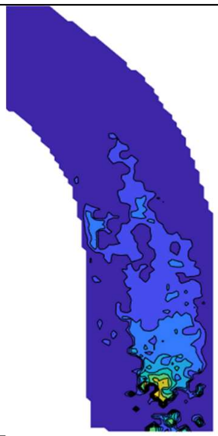 | 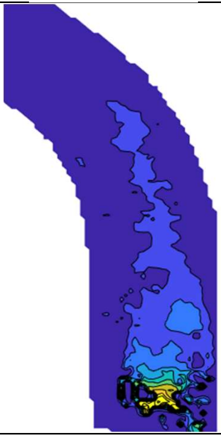 | 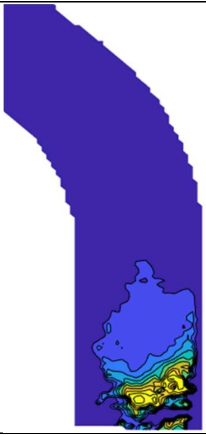 |

**Table S13: TKE PIV - Counterclockwise flow**

| | 1 L/min | 1.5 L/min | 2 L/min | 2.5 L/min |
|---|---|---|---|---|
| Distal section | 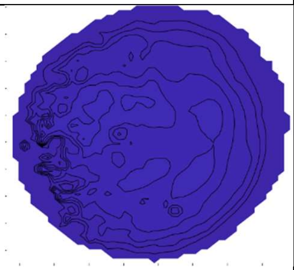 | 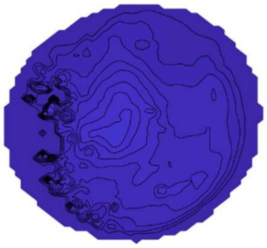 | 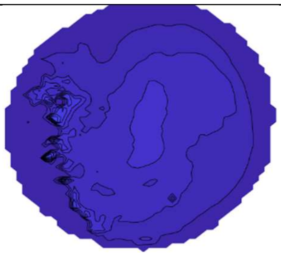 | 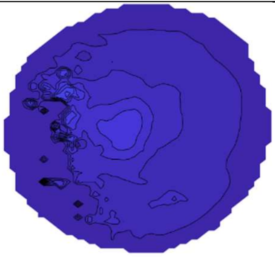 |
| Middle section | 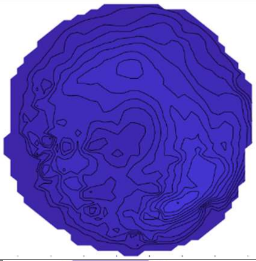 | 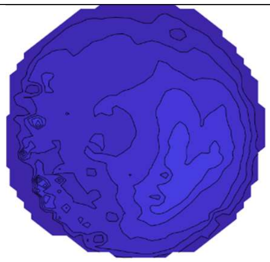 | 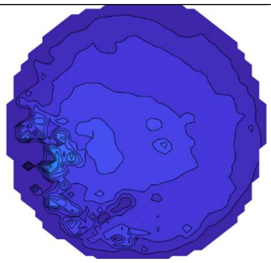 | 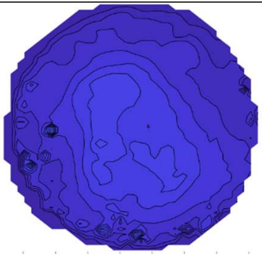 |
| Proximal section | 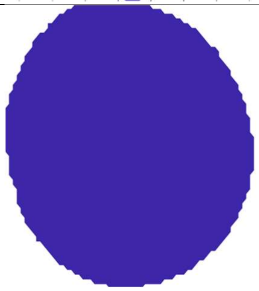 | 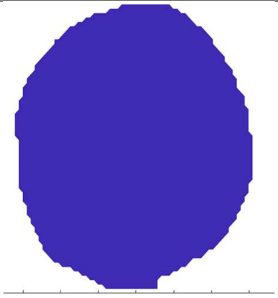 | 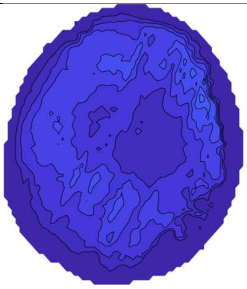 | 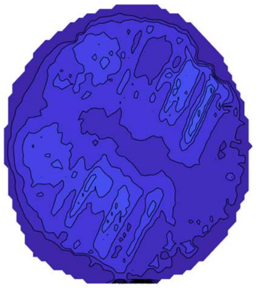 |
| Sagittal section | 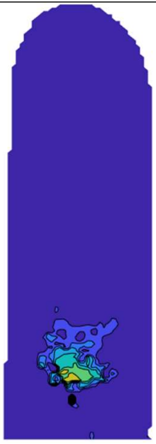 | 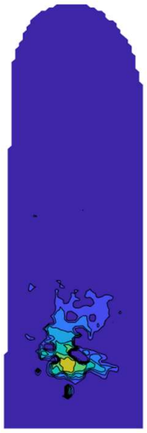 | 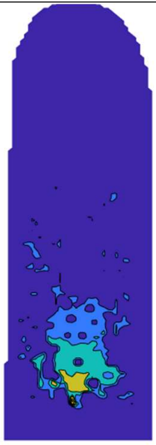 | 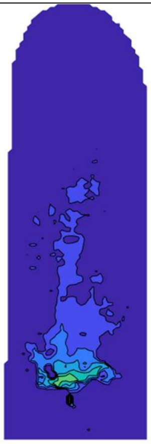 |
| Coronal section | 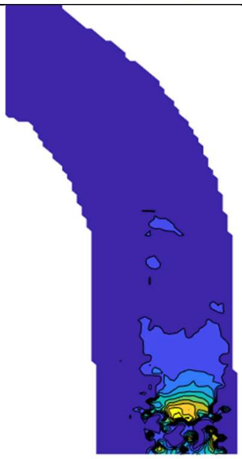 | 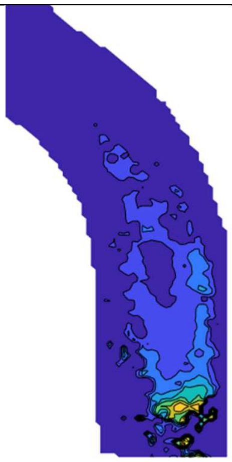 | 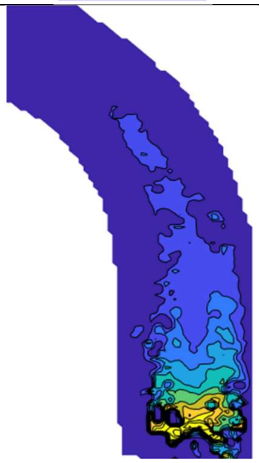 | 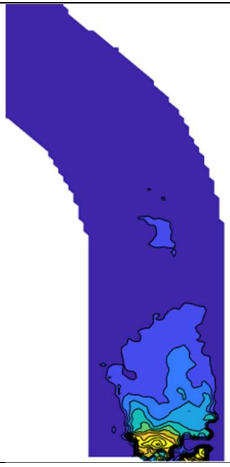 |
